# RNAi inhibition of feruloyl CoA 6′-hydroxylase reduces scopoletin biosynthesis and post-harvest physiological deterioration in cassava (*Manihot esculenta* Crantz) storage roots

**DOI:** 10.1007/s11103-017-0602-z

**Published:** 2017-03-18

**Authors:** Shi Liu, Ima M. Zainuddin, Herve Vanderschuren, James Doughty, John R. Beeching

**Affiliations:** 1 Department of Biology and Biochemistry, University of Bath, Bath, BA2 7AY UK; 2 Department of Biology, Plant Biotechnology, Eidgenössische Technische Hochschule (ETH) Zurich, Universitätstrasse 2, 8092 Zurich, Switzerland; 3 Research Center for Biotechnology, Indonesian Institute of Sciences, Complex CSC-LIPI Jl. Raya Bogor Km 46, Cibinong, Bogor, West Java 16911 Indonesia; 4 Plant Genetics, AgroBioChem Department, Gembloux Agro-BioTech, University of Liège, 4000 Liège, Belgium

**Keywords:** *Arabidopsis thaliana*, *Manihot esculenta*, Feruloyl CoA 6′-hydroxylase, Post-harvest physiological deterioration, RNA interference, Scopoletin

## Abstract

Cassava (*Manihot esculenta* Crantz) is a major world crop, whose storage roots provide food for over 800 million throughout the humid tropics. Despite many advantages as a crop, the development of cassava is seriously constrained by the rapid post-harvest physiological deterioration (PPD) of its roots that occurs within 24–72 h of harvest, rendering the roots unpalatable and unmarketable. PPD limits cassava’s marketing possibilities in countries that are undergoing increased development and urbanisation due to growing distances between farms and consumers. The inevitable wounding of the roots caused by harvesting triggers an oxidative burst that spreads throughout the cassava root, together with the accumulation of secondary metabolites including phenolic compounds, of which the coumarin scopoletin (7-hydroxy-6-methoxy-2H-1-benzopyran-2-one) is the most abundant. Scopoletin oxidation yields a blue-black colour, which suggests its involvement in the discoloration observed during PPD. Feruloyl CoA 6′-hydroxylase is a controlling enzyme in the biosynthesis of scopoletin. The cassava genome contains a seven membered family of feruloyl CoA 6′-hydroxylase genes, four of which are expressed in the storage root and, of these, three were capable of functionally complementing Arabidopsis T-DNA insertion mutants in this gene. A RNA interference construct, designed to a highly conserved region of these genes, was used to transform cassava, where it significantly reduced feruloyl CoA 6′-hydroxylase gene expression, scopoletin accumulation and PPD symptom development. Collectively, our results provide evidence that scopoletin plays a major functional role in the development of PPD symptoms, rather than merely paralleling symptom development in the cassava storage root.

**Electronic supplementary material:**

The online version of this article (doi:10.1007/s11103-017-0602-z) contains supplementary material, which is available to authorized users.

## Introduction

Cassava (*Manihot esculenta* Crantz), a root crop belonging to the Euphorbiaceae, is the major staple food crop for more than 800 million people throughout the humid tropics (Nassar et al. 2007; Sánchez et al. 2013). It is particularly important to the sustainable livelihoods of resource-poor farmers in many parts of Africa, Asia and America due to cassava’s ability to produce acceptable yields on marginal soils with minimal inputs, and to its drought tolerance (Burns et al. 2010; El-Sharkawy 2004, 2012; Xu et al. 2013). In addition, under good commercial agronomic conditions, cassava can produce yields of over 90 tonnes per hectare, whilst on research stations yields of up to 90 tonnes have been achieved, making it a valuable commodity for the production of animal feed, starch and, increasingly, bioethanol (Beeching 2013; Fermont et al. 2009). Like all crops, cassava is susceptible to a range of insect, microbial and viral pests (Bellotti 2002; Calvert and Thresh 2002; Hillocks and Wydra 2002). However, a constraint that is virtually unique to the storage roots is their rapid deterioration upon harvesting (Beeching et al. 1998a). This post-harvest physiological deterioration (PPD) causes discoloration and organoleptic changes in the roots within 24–72 h of harvest, which renders them unpalatable and unmarketable, leading to price discounts and wastage. As a result, the roots must be either consumed locally or processed into a more stable form soon after harvesting to prevent losses. PPD particularly impacts larger industrial processing plants at a distance from producers, and urban customers may end up choosing more reliable, but sometimes imported, sources of carbohydrate (Uarrota and Maraschin 2015).

Strategies to reduce wastage due to PPD have not proved satisfactory. In village communities, cassava can be left in the ground and harvested only when needed, but this ties up otherwise productive land. Alternatively, the roots can be sealed with wax or frozen; however, for such a low value commodity, this solution is only appropriate for urban elites or expatriates prepared to pay a premium for their traditional foods. While some cassava material has been identified with reduced deterioration properties, breeding approaches have not proved successful due the polygenic nature of the trait and its correlation with the desirable high dry-matter content (Cortés et al. 2002; Morante et al. 2010). Biotechnological approaches can provide useful insights into the nature of PPD and, additionally, may lead to means through which the shelf-life of the harvested roots could be usefully extended; however, they are relatively untried (Beeching et al. 1998b; Sayre et al. 2011).

The visible symptoms of post-harvest physiological deterioration (PPD) in cassava roots are the blue-black discoloration that starts in the vascular tissue and with time spreads more extensively through much of the root, together with a fluorescence under UV light that is detectable prior to and during the discoloration. These symptoms are due to the accumulation of phenolic compounds and their oxidation by reactive oxygen species (ROS). There is an oxidative burst in the roots shortly after harvest: within 15 min superoxide is detectable, which declines 8–10 h later, while hydrogen peroxide is detectable within 3 h, peaks at 24 and gradually declines thereafter. While the expression of anti-oxidant genes and proteins for superoxide dismutase (SOD), catalase (CAT) and ascorbate peroxidase (APX) occurs, these events are either too late or inadequate to attenuate the ROS, and as a result, the oxidative burst spreads through the root (Owiti et al. 2011; Reilly et al. 2007, 2004; Vanderschuren et al. 2014). Various secondary metabolites are synthesised post-harvest in the cassava root, of which the principal is the coumarin scopoletin (7-hydroxy-6-methoxy-2H-1-benzopyran-2-one) that fluoresces under UV. Peroxidase mediated oxidation of scopoletin by hydrogen peroxidase yields a blue-black colour, which is probably responsible for much of the discoloration of PPD (Bayoumi et al. 2010; Buschmann et al. 2000a, b; Gutierrez 1995).

These data suggest that by altering the biosynthesis of anti-oxidant enzymes and through interfering with the biosynthesis of scopoletin, insights could be gained into the relationships between ROS, scopoletin and PPD. Transgenic cassava roots in which SOD and CAT were co-overexpressed, or cyanide-insensitive alternative oxidase (AOX) was over-expressed, showed reduced deterioration responses, though, in the case of AOX, the roots had reduced biomass (Xu et al. 2013; Zidenga et al. 2012). The use of isotope labelled intermediates to examine three alternative pathways to scopoletin biosynthesis confirmed that the pathway via ferulate contributes most of scopoletin accumulated in cassava storage roots, while only approximately 20% of scopoletin and its glycine, scopolin, was accumulated via the two minor pathways (Bayoumi et al. 2008b). The principal pathway (via ferulate) is also the dominant one in *Arabidopsis*, as T-DNA insertion mutants in the F6′H1 (feruloyl CoA 6′-hydroxylase 1) gene, which regulates the conversion of ferulate to 6′-hydroxylate in the biosynthesis of scopoletin, effectively prevented scopoletin accumulation in the roots (Kai et al. 2008, 2006). These results suggest a strategy by which to modify scopoletin biosynthesis in cassava. Therefore, in this paper we examine the effects of down-regulating MeF6′H gene expression, using RNAi, on scopoletin accumulation and on the PPD response in cassava storage roots.

## Materials and methods

### Chemical and plant materials

Chemicals used in this study were obtained from Sigma–Aldrich (St Louis, MO) unless otherwise stated. *Arabidopsis thaliana* T-DNA insertion mutants, Salk_129938 and Sail_001252_A10, were obtained from the Nottingham Arabidopsis Stock Centre (NASC) (Alonso et al. 2003). Arabidopsis (mutant or wild-type Col-0) plants were grown in a controlled environment suite (Weiss–Gallenkamp, Loughborough, United Kingdom) at 21 °C, 60% relative humidity, and 16 h of daylight a day. In vitro Arabidopsis was grown in square Petri dishes on 1.6% w/v phyto-agar medium with 4.4 g/L Murashige and Skoog salts, and with Gamborg’s vitamins (Melford, Ipswich, United Kingdom), 10 g/L sucrose, and 0.5 g/L MES (Melford, Ipswich, United Kingdom). These plates were placed vertically to encourage the growth of roots.

Cassava plants (cv. 60444) were grown in a glasshouse with supplemented lighting from 400 W Philips high-pressure sodium lights at 30 °C during the day and 17 °C during the night. The storage roots were harvested after 6–7 months growth.

### Nucleic acid purification

A small rosette leaf from Arabidopsis was placed in a sterile 1.5 mL microfuge tube with 200 μL of DNA extraction buffer (0.14 M d-Sorbitol, 0.22 M Tris–HCl (pH = 8.0), 0.022 M EDTA (pH = 8.0), 0.8 M NaCl, 0.8% CTAB, and 0.1% n-Laurylsarcosine). The leaf was homogenised with a pillar drill and incubated at 65 °C for 5 min. The homogenate was vigorously vortexed with 100 μL of chloroform and centrifuged in a microfuge at 13,000 rpm for 5 min. The aqueous supernatant was transferred to a fresh microfuge tube and mixed with 150 μL 100% isopropanol. The mixture was incubated at room temperature for 15 min and centrifuged at 13,000 rpm for 20 min to pellet the DNA. The supernatant was discarded and the pellet was washed once with 500 μL of 70% EtOH. The pellet was allowed to air-dry for 10 min and re-suspended in 50 μL of MilliQ water.

Arabidopsis total RNA was extracted with a SV total RNA isolation system (Promega, Madison, WI) following the manufacturer’s instructions. Cassava total RNA was extracted from the storage roots at different stages of PPD development (Chang et al. 1993). The extracted total RNA was digested with Turbo DNase (Thermo Fisher, Waltham, MA) following the manufacturer’s instructions to remove carryover DNA, and purified with chloroform. 150 ng of total RNA was used in cDNA synthesis with a High-Capacity cDNA Reverse Transcription Kit (Applied Biosystems, Foster City, CA) following the manufacturer’s instructions.

### Retrieval and analysis of gene expression

Genes sequences were identified in and retrieved from Phytozome (https://phytozome.jgi.doe.gov/pz/portal.html) (Bredeson et al. 2016; Goodstein et al. 2012) using TBLASTN with default parameters (Altschul et al. 1990; Gertz et al. 2006; Goodstein et al. 2012; Prochnik et al. 2012). Phylogenic analysis of genes was carried out using Geneious R8 (Kearse et al. 2012).

### Quantification of MeF6′H genes expression

To verify the expression of MeF6′H genes, RT-PCR was carried out with cDNA synthesized from cassava root RNA. Primers were designed for every MeF6′H gene: MeF6′H1 (For.: GGTACTCGATGGAGTCAAGGAC; Rev.: CGCCGACAGTAAGATTAGGATTG); MeF6′H2 (For.: GAGCGTAGAGCATTGTGTGATTA; Rev.: CTTCCTGAAGAAATGCTTGACG); MeF6′H3 (For.: TGGGCATCAAAAGCCTTCCTATA; Rev.: GCAACTCATGGTTTCTTTGGAC); MeF6′H4 (For.: CAAAGAGCTTTCTTCCACCAACAATA; Rev.: GTTGATCACAAGGGATCCTTCAATT); MeF6′H5 (For.: GCTCCAGCAATGGCAGTG; Rev.: ACGTTCAGTTTCTCCATGAGTGT); MeF6′H6 (For.: GCATCAAAAGCCTTCCTCGC; Rev.: CACAGCAGCTGGAACCCC); MeF6′H7 (For.: CTTTAGTCCTGATGCAGAGAAG; Rev.: AACAGTCTTCTTGCCATCG). Each primer pair underwent a series of PCR reactions over a range of annealing temperatures (55–65 °C) to identify optimum conditions.

### Arabidopsis mutant verification

The seeds of T-DNA insert mutant lines obtained from NASC were verified by PCR amplification to confirm homozygous lines with the T-DNA insert in both alleles. The seeds were sown on compost and DNA was extracted from the rosettes of the seedlings. The absence of the wild-type Arabidopsis F6′H1 (Phytozome ID: At3g13610) gene and the presence of the T-DNA insert was verified by genotyping PCR with line-specific primers and a T-DNA left border-specific primer as recommended by the Salk institute (http://signal.salk.edu/cgi-bin/tdnaexpress). Seedlings showing the presence of the T-DNA insert and the absence of wild-type F6′H1 gene were considered to be homozygous mutants.

### Arabidopsis and cassava transformation

DNA was cloned into either the pKannibal vector (Helliwell and Waterhouse 2003) for hairpin RNAi constructs and/or a Gateway-modified pCAMBIA 1305.1 vector (GenBank accession: AF354045) for gene expression studies in transformed Arabidopsis and cassava using Gateway cloning methods (Karimi et al. 2002). The Gateway^®^ Vector Conversion System (Invitrogen, Carlsbad, CA), Gateway^®^ BP Clonase™ II (Invitrogen, Carlsbad, CA), and Gateway^®^ LR Clonase™ II (Invitrogen, Carlsbad, CA) were used following the manufacturer’s instructions. The RNAi and expression constructs were driven by either the constitutive CaMV 35 S or the root-specific Patatin promoter (Bull 2011; Page 2009). The RNAi construct was amplified from the first exon of MeF6′H3 (For.: CCAACACTTGCAGAATCAGCC; Rev.: ATTAGCCTCGTCGTCGGAGA). Full attB sequences (For.: GGGGACAAGTTTGTACAAAAAAGCAGGCT; Rev.: GGGGACCACTTTGTACAAGAAAGCTGGGT) and necessary restriction sites were added to the cloned MeF6′H genes and the RNAi constructs via PCR reactions.

*Agrobacterium*-mediated transformation was used for both Arabidopsis and cassava (Bevan 1984; Hoekema et al. 1983): the floral dipping method for Arabidopsis, following the standard protocol, and the friable embryogenic calli (FEC) method was used for cassava transformation (Bull et al. 2009; Clough 2005; Clough and Bent 1998; Zainuddin et al. 2012).

### PPD induction and quantification of discoloration

Storage roots of cassava were harvested and cleaned, with both proximal and distal ends removed. The roots were cut into approximately 1 cm thick slices and placed in sterile Petri dishes on a piece of dry filter paper (Whatman, Maidstone, United Kingdom). The root samples were kept in a controlled environment suite at 24 °C, relative humidity 50%, over a time-course of 5 days to develop discoloration.

The dried surface of the root samples was removed to expose a fresh section. This was photographed at a consistent exposure and the PPD discoloration level of the slice quantified with a MatLab-based software to determine the PPD score (Vanderschuren et al. 2014). The greyscale scores were standardised with the greyscale of the filter paper; therefore, fresh samples without PPD showed scores above zero.

### Scopoletin quantification

Roots from 4-week old Arabidopsis grown vertically on MS-MES plates (Kai et al. 2008; Page 2009) were washed and blotted dry before being ground in 1.5 mL microfuge tubes using sterile plastic pestles in liquid nitrogen. Cassava storage root samples were ground in a sterile pestle and mortar with liquid nitrogen.

15–30 mg of Arabidopsis root sample or 200–700 mg of cassava root sample was transferred to a sterile 2 mL screw-cap microfuge tube (Molecular BioProducts, San Diego, CA). 2.0 mL of Extraction solvent (100 ng/mL 4-MU in HPLC-grade MeOH) was added to the root powder and the tube was incubated at room temperature on a vertical rotor (Labinco B. V., Breda, Netherlands) at 10 rpm for 16 h. The tube was then centrifuged at 10,000 rpm for 5 min to pellet the debris. The supernatant was filtered through a Minisart 0.25 μm NY syringe filter (Sartorius, Gottingen, Germany), transferred to a 2 mL screw-cap microfuge tube (Molecular BioProducts, San Diego, CA) and concentrated in a Savant SpeedVac vacuum concentrator (Thermo Fisher, Waltham, MA) for approximately 3.5 h until all the solvent had evaporated. 200 μL (for Arabidopsis root sample) or 400 μL (for cassava root sample) of Re-dissolving solvent (1 μg/mL scoparone in HPLC-grade MeOH) was added to the tube and incubated at 4 °C for 48 h to dissolve the pelleted extract. The re-dissolved sample was vortexed and centrifuged at 13,000 rpm for 5 min to pellet any contaminating particles. 150 μL of supernatant was transferred to a 300 μL screw-cap vial (Waters, Milford, MA). The sample was quantified by LC-MS with a Daltonics micrOTOF spectrometer (Bruker, Billerica, MA) using Electrospray Ionisation (ESI) with a reverse phase C18 coated column. A mobile phase of gradient MeOH/water was used (Kai et al. 2006). A standard scopoletin sample (Sigma–Aldrich, St Louis, MO) was used to draw the standard curve.

### Quantification of MeF6′H genes expression

The reaction was set up in a MicroAmp Fast Optical 96-Well Reaction Plate with Fast SYBR Green mastermix, and sealed with MicroAmp Optical Adhesive Film, following the manufacturer’s instructions (Applied Biosystems, Foster City, CA). Primers to quantify the MeF6′H genes were (For.: TGGGTTCATGTTCCTCCCAT; Rev.: CTCCTATATCGACCATTGCTGAGTA) and those to quantify the ubiquitin 10 reference gene (NCBI Accession number DV441403) were (For.: CACCGGATCAGCAAAGGCTTA; Rev.: CAGACACACAGATCAAAGCAGC) (Moreno et al. 2011). The StepOne Software v2.3 (Applied Biosystems, Foster City, CA) was used to operate the qRT-PCR reactions, and the amplification efficiencies of the primers were tested with serially diluted cDNA samples and a standard curve method to ensure good quantification ability (R-squared > 0.95) and optimal amplification efficiencies (90–110%). The target genes were quantified using the ΔΔCt method (Livak and Schmittgen 2001).

## Results

### Cassava possesses a small family of feruloyl 6′-hydroxylase genes

**Fig. 1:**
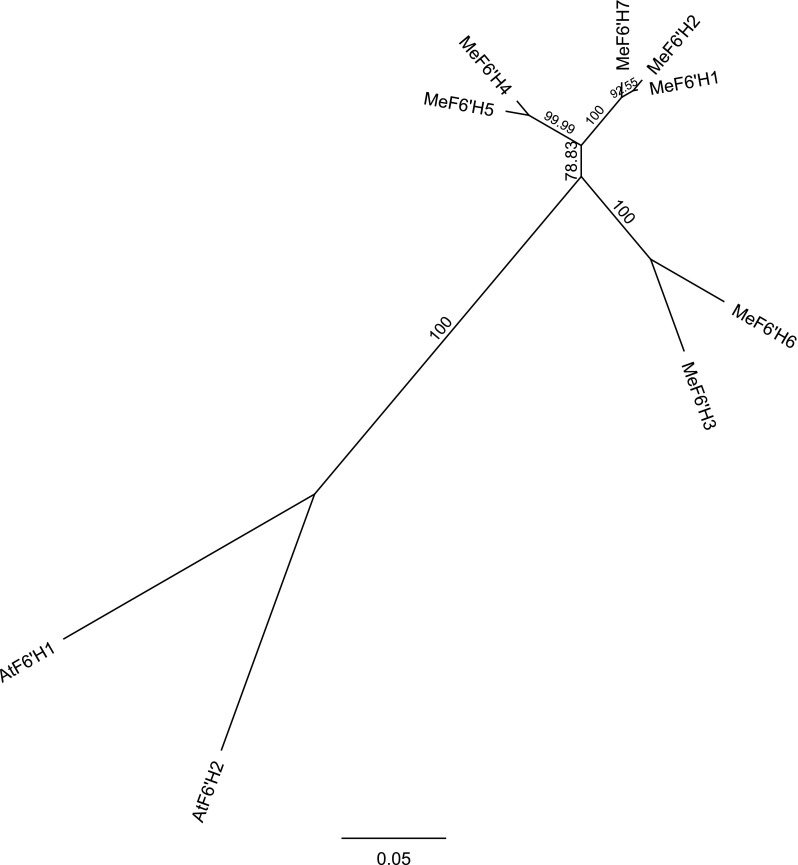
Phylogenetic tree (with bootstrapping) showing the similarities between Arabidopsis F6′H1 and cassava candidate genes. This tree is based on amino acid sequences. A neighbour joining (NJ) method is used and bootstrapping is repeated 10,000 times. F6′H1 and F6′H2 are referred to as AtF6′H1 and AtF6′H2 in this phylogenetic tree in order to make their Arabidopsis origin explicit

Seven cassava genes showing high similarity to the predicted amino acid sequence of Arabidopsis F6′H1 (Phytozome ID: At3g13610) were identified in the cassava genome: *MeF6′H1* (cassava4.1_010291 m.g, accession number: KY078747), *MeF6′H2* (cassava4.1_010292 m.g, KY078746), MeF6′H3 (cassava4.1_010376 m.g, KY078745), *MeF6′H4* (cassava4.1_010381 m.g), *MeF6′H5* (cassava4.1_027567 m.g), *MeF6′H6* (cassava4.1_030526 m.g), and *MeF6′H7* (cassava4.1_033240 m.g) (supplementary data, table S1 and S2). This family of putative MeF6′H genes is considerably larger than that of Arabidopsis, which has only a second feruloyl CoA 6′-hydroxylase gene in addition to *F6′H1, F6′H2* (Phytozome ID: At1g55290), that plays a negligible role in scopoletin biosynthesis (Kai et al. 2008). An un-rooted phylogenetic tree revealed that the seven cassava genes clustered close to each other but separately from the two Arabidopsis genes; certainly, no one cassava gene clustered sufficiently close to the *F6′H1* to suggest that it might be a functional feruloyl CoA 6′-hydroxylase (Fig. 1).

**Fig. 2:**
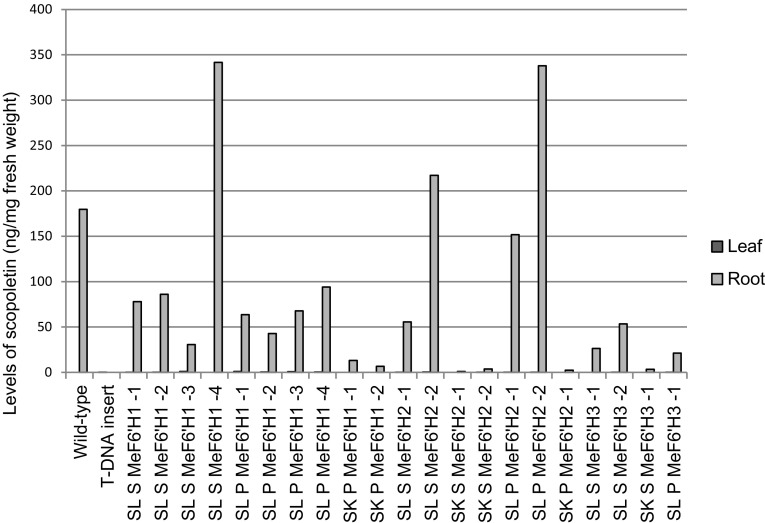
The scopoletin biosynthetic complementation in Sail_1252_A10 and Salk_129938 F6′H1 T-DNA insert mutants of Arabidopsis with different cassava candidates and promoters. Data show scopoletin levels from LC-MS assays. Notice that in most samples the leaf scopoletin levels are too low to be detected. Salk-CaMV35S-MeF6′H1 transgenesis failed. SL: Sail_1252_A10; SK: Sail_129938; S: CaMV 35 S promoter; P: StPAT promoter; the number at the end of the names indicates the particular transgenic line tested. Two replicates were tested for each transgenic line. Therefore, standard deviations are not available

The expression of all seven cassava genes was tested by RT-PCR using gene-specific primers, which confirmed the active expression of *MeF6′H1, MeF6′H2, MeF6′H3*, and *MeF6′H5* only in wounded cassava storage-root tissue (1 DAH onwards), but not in fresh roots (supplementary data, figure S1). In order to confirm which of these four genes encodes a functional feruloyl 6′-hydroxylase enzyme they were each fused to either the root-specific StPAT (Jefferson et al. 1990; Page 2009) or the constitutive CaMV 35 S promoter and transformed into homozygous Arabidopsis T-DNA insertion mutants of F6′H1, Salk_129938 and Sail_001252_A10. Relative scopoletin levels were assayed in the roots and leaves of several lines from these transformation events (Fig. 2). While scopoletin levels in leaves were barely detectable, these data identified several transgenic lines for three of the cassava genes, *MeF6′H1, MeF6′H2*, and *MeF6′H3*, that were able to complement the T-DNA insertion mutation and accumulate high levels of scopoletin in their roots. In several lines this accumulation was in excess to that observed in the Arabidopsis wild-type. The variation in scopoletin accumulation levels in different transgenic lines from the same construct is probably due to variation in transgene expression levels. These data confirm that at least three members of the cassava gene family possess functional feruloyl CoA 6′-hydroxylase activity.

### RNAi transgene caused a few morphological differences in transgenic cassava plants

The growth of stems of the wild-type and RNAi transgenic cassava were measured and there was no significant difference in the rate of stem elongation (data not shown). However, RNAi transgenic cassava grew leaves that showed obviously different shape compared to that of wild-type plants (figure S2). The transgenic plants often grew leaves that showed broader lobes, and they were often curled and folded. The fluorescence of wild-type and RNAi transgenic cassava roots under ultraviolet light were also qualitatively compared (figure S3) and showed similar pattern of behaviour. Quantitative assays on the strength of fluorescence were unavailable.

### MeF6′H RNAi reduced the PPD response in transgenic cassava roots

Comparison of the nucleotide sequences of the four MeF6′H genes (i.e. *MeF6′H1, MeF6′H2, MeF6′H3, MeF6′H5*) expressed in the cassava roots showed particularly high levels of identity within the first exon, in fact there were long stretches that showed absolute identity that were also shared beyond these four to the other members of the gene family. Therefore, a sequence of 471 nucleotides with longest possible conservative sequence runs was chosen and amplified from the cloned cDNA of *MeF6′H3* to make an RNAi construct. The overall similarity of this sequence in the first exon scored 79.76–81.95% compared to the corresponding parts of gene *MeF6′H1, MeF6′H2*, and *MeF6′H5*, respectively (alignment of RNAi and MeF’Hs in supplementary data). Such a sequence has a high probability to target and thereby prevent the expression of, not only the gene to which it was designed, but also other family members, thereby exploiting the potential off-target effects of a construct designed to a single gene (Jackson et al. 2003).

**Fig. 3:**
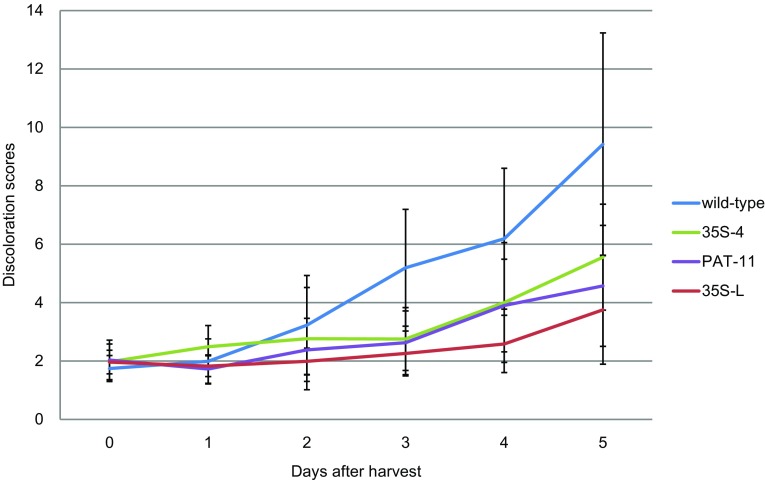
The discoloration development of the three selected RNAi T-DNA insert mutants is quantitatively compared to the wild-type cassava. This chart shows the relative discoloration of samples in a time-course of 5 days. Data are mean discoloration scores +/− standard deviation from between seven and 17 independent replicates

**Fig. 4:**
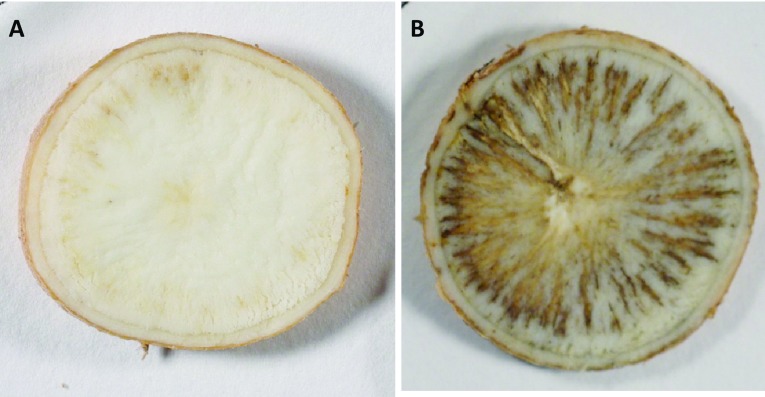
The discoloration development of an RNAi T-DNA insert mutant is graphically compared to the wild-type cassava. **a** A representative 35S-L root sample shows little PPD discoloration on 5DAH. **b** A representative wild-type root sample shows significant PPD discoloration on 5DAH. DAH: days after harvest

Two RNAi constructs driven by either a root-specific StPAT promoter or a constitutive CaMV 35S promoter were used to transform wild-type cassava (cv. 60444). Nine transgenic lines were obtained and the discoloration of their storage roots was quantified over a time-course of 5 days from harvest. Three of these lines, 35S-4, 35S-L and PAT-11, showed significant (P < 0.05) reductions in PPD discoloration response compared to the wild-type control (Figs. 3, 4). Therefore, these three lines were chosen to be taken forward for further analyses.

### MeF6′H RNAi inhibited the expression of the feruloyl CoA 6′-hydroxylase gene family in transgenic cassava roots

**Fig. 5:**
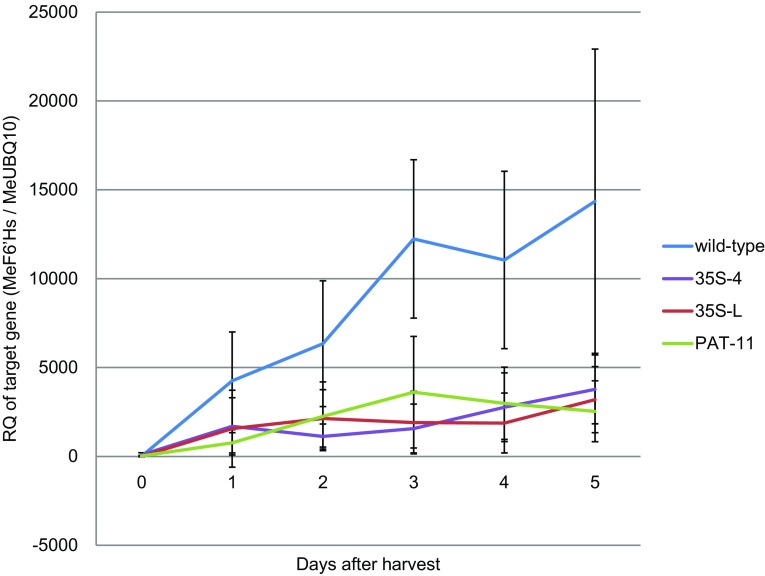
The expression levels of the MeF6′H gene family in wild-type cassava and three selected RNAi transformant lines. Data are mean relative quantification of MeF6′Hs mRNA +/− standard deviation in wild-type and three RNAi transformant lines over a time-course of 5 days as determined by qRT-PCR from between five to 11 independent samples

The high sequence similarity between family members and the target size-restrictions for RT-qPCR did not permit the design of gene-specific primers. Therefore, the expression of the four expressed *MeF6′H* gene family members (and also *MeF6′H7* if it were expressed) was measured in cassava roots over a 5 day post-harvest time-course using generic primers designed to amplify all four members. The three RNAi transgenic lines that showed significant reduction in the visible PPD response also showed significant reduction in feruloyl CoA 6′-hydroxylase mRNA accumulation on days 3–5 after harvest (P < 0.01, except 35S-4 on 5 DAH, P < 0.05) compared to wild-type (Fig. 5).

While RNAi silencing in plants tolerates less mismatching than in animal cells (Jackson et al. 2003; Schwab et al. 2006), this down-regulation of *MeF6′H* expression in the transgenic cassava confirms that the selection of part of first exon of the gene MeF6′H3, which contains a longest possible stretch of identical nucleotides for the RNAi constructs, enabled it to target multiple genes.

### MeF6′H RNAi reduced scopoletin accumulation in transgenic cassava roots

**Fig. 6:**
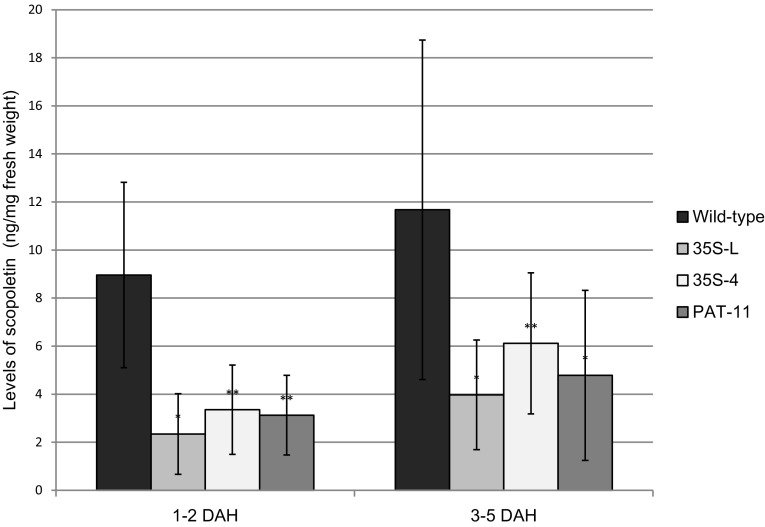
The accumulation of scopoletin during the early (1–2 DAH) and the late (3–5 DAH) stage of PPD in three selected RNAi mutants and wild-type cassava. Data are mean scopoletin levels (ng/mg fresh sample) +/− standard deviation as determined by LC-MS assays from between three to six independent samples. T-test was used to verify significant difference between the wild-type cassava (TMS 60444) and the RNAi lines at P < 0.01 (one *asterisk*) or P < 0.05 (two *asterisk*s)

Feruloyl-CoA 6′-hydroxylase catalyses a controlling step in the most important scopoletin biosynthetic pathway in cassava (Bayoumi et al. 2008a; Kai et al. 2008). Above, we have functionally confirmed the feruloyl-CoA 6′-hydroxylase identity of three cassava genes; therefore, the inhibition of MeF6′H expression in the RNAi transgenic plants could affect the accumulation of scopoletin in the storage roots. At harvest the levels of scopoletin in all roots, both transgenic and wild-type controls, were barely detectable. However, over a time-course of 5 days post-harvest, the levels rose in all plants, though significantly less (P < 0.05) in the three selected transgenic lines compared to the wild-type control (Fig. 6), thereby confirming the inhibition of the MeF6′H gene family led to reduced scopoletin accumulation in cassava roots.

## Discussion

Cassava possesses a feruloyl CoA 6′-hydroxylase (MeF6′H) gene family with seven members of which four are expressed in storage root tissue. Cassava is an allotetraploid (Sakurai et al. 2007; Salehuzzaman et al. 1993); therefore, a family larger than that of two found in Arabidopsis is not surprising. In Arabidopsis only F6′H1 plays a dominant role in scopoletin synthesis (Kai et al. 2008) and in cassava the expression data also suggest that several family members are silent. Functional complementation of T-DNA insertion mutants of F6′H1 proves that at least three of the MeF6′H genes coded for enzymes with feruloyl 6′-hydroxylase activity. However, it must be noted that in the small number of independent transgenic lines tested for each gene the accumulation of scopoletin varied considerably between them, presumably caused by position effects due to insertion into transcriptionally active or quiescent regions of the genome (De Bolle et al. 2003).

Due to regions of high sequence identity in the MeF6′H genes, it proved possible to design an RNAi construct with the potential to target several, if not most, members of the MeF6′H gene family. This construct led to reduced MeF6′H gene expression, reduced scopoletin accumulation and reduced PPD symptoms in the storage roots of transgenic cassava after harvest. However, complete inhibition was not achieved. Linear regression showed that there was significant correlation between the expression of MeF6′Hs and the levels of discoloration, and between the levels of MeF6′H expression and the scopoletin accumulation (Supplementary data, figure S4 and S5). Nonetheless, this is very strong evidence that scopoletin plays a significant role in the development of PPD symptoms; until now the accumulation of scopoletin has largely been perceived as paralleling symptom development rather than playing a functional role in causing the symptoms *per se* (Bayoumi et al. 2010; Buschmann et al. 2000a).

A striking observation was the dramatic increase in MeH6′H gene-family expression over the PPD time-course in wild-type cassava, from barely detectable levels to approximately 10,000-fold higher in 5 days. This is accompanied by a concomitant increase in scopoletin levels, which confirms that the synthesis of scopoletin is triggered de novo upon harvest. In the RNAi plants MeF6′H genes expression was significantly reduced compared to controls as were the resultant scopoletin accumulation and PPD symptoms. This leakage in, rather than complete inhibition of, MeH6′H gene expression in the RNAi plants, could be due to the small sequence differences present in the target-sequences of the various gene family members for the RNAi preventing complete base-pairing to and the resultant degradation of the transcripts. Alternatively, as a universal gene inactivating technique, RNAi may itself be inadequate for a complete gene silencing. In Arabidopsis, double-stranded RNA hairpins confer varied efficiency when silencing different genes, implying that this technique may not be efficient for every gene (Wesley et al. 2001). A statistical analysis on 429 cases of published mammalian cell RNA interference data showed, that in approximately 40% of cases the expression of the target genes was reduced by less than 50% (Munkacsy et al. 2016). Although mammalian cells are very different from plants, this suggests an inadequacy of double-stranded RNA as a tool for complete gene silencing. Therefore, complete inhibition of the expression of MeH6′H gene family may only be possible through multiple RNAi constructs or multiple gene-editing events, each designed to a specific MeF6′H gene. Such plants would permit determining whether or not scopoletin is the only or just the major contributor to the symptoms of PPD, and whether the resultant extended shelf-life could be economically useful. In addition to its oxidation producing the symptoms of PPD, scopoletin acts as an anti-oxidant and its major biological role is as an anti-microbial phytoalexin (Gnonlonfin et al. 2012; Gutiérrez-Mellado et al. 1996). Therefore, totally blocking the biosynthesis of scopoletin may have implications for defence of the crop against pathogens.

## Electronic supplementary material

Below is the link to the electronic supplementary material.

Supplementary material 1 (PPTX 449 KB)

Supplementary material 2 (PPTX 1943 KB)

Supplementary material 3 (PPTX 3042 KB)

Supplementary material 4 (PPTX 379 KB)

Supplementary material 5 (PPTX 379 KB)

Supplementary material 6 (PDF 71 KB)

Supplementary material 7 (DOCX 20 KB)

## Abbreviations

AOX: Cyanide-insensitive alternative oxidase
APX: Ascorbate peroxidase
AtF6′H: *Arabidopsis thaliana* feruloyl CoA 6′-hydroxylase
CAT: Catalase
CaMV: Cauliflower mosaic virus
DAH: Days after harvest
EtOH: Ethanol
For.: Forward;
F6′H: Feruloyl CoA 6′-hydroxylase
FW: Fresh weight
MeF6′H: *Manihot esculenta* feruloyl CoA 6′-hydroxylase
LC-MS: Liquid chromatography–mass spectrometry
MeOH: Methanol
MES: 2-(N-morpholino) ethanesulfonic acid
MS: Murashige & Skoog medium
NASC: Nottingham Arabidopsis stock centre
NJ: Neighbour joining
PPD: Post-harvest physiological deterioration
qRT-PCR: Quantitative reverse transcription polymerase chain reaction
Rev.: Reverse
RNAi: RNA interference
ROS: Reactive oxygen species
SOD: Superoxide dismutase
UV: Ultraviolet

## References

[CR1] Alonso JM (2003). Genome-wide insertional mutagenesis of *Arabidopsis thaliana*. Science.

[CR2] Altschul SF, Gish W, Miller W, Myers EW, Lipman DJ (1990). Basic local alignment search tool. J Mol Biol.

[CR3] Bayoumi SA, Rowan MG, Blagbrough IS, Beeching JR (2008). Biosynthesis of scopoletin and scopolin in cassava roots during post-harvest physiological deterioration: the E-Z-isomerisation stage. Phytochemistry.

[CR4] Bayoumi SAL, Rowan MG, Blagbrough IS, Beeching JR (2008). Investigation of scopoletin biosynthesis during post-harvest physiological deterioration in cassava roots using stable isotopic labelling. J Pharm Pharmacol.

[CR5] Bayoumi SAL, Rowan MG, Beeching JR, Blagbrough IS (2010). Constituents and secondary metabolite natural products in fresh and deteriorated cassava roots. Phytochemistry.

[CR6] Beeching JR (1998a) An abiotic stress response in cassava: Post-harvest physiological deterioration Annual Symposium of the Plant Protein Club at York, “Plant Proteins in Abiotic Stress Responses” York, Sept 27 Oct 1

[CR7] Beeching JR (2013) *Manihot esculenta* (Cassava). eLS:1–7. doi:10.1002/9780470015902.a0023710

[CR8] Beeching JR, Han Y, Gómez-Vásquez R, Day RC, Cooper RM (1998). Wound and defense responses in cassava as related to post-harvest physiological deterioration. Recent Adv Phytochem.

[CR9] Bellotti A, Hillcocks RJ, Thresh JM, Bellotti A (2002). Arthropod pests. Cassava: biology, production and utilization.

[CR10] Bevan M (1984). Binary *Agrobacterium* vectors for plant transformation. Nucleic Acids Res.

[CR11] Bredeson JV (2016). Sequencing wild and cultivated cassava and related species reveals extensive interspecific hybridization and genetic diversity. Nat Biotechnol.

[CR12] Bull SE (2011) Study of post-harvest physiological deterioration in transgenic cassava., University of Bath

[CR13] Bull SE, Owiti JA, Niklaus M, Beeching JR, Gruissem W, Vanderschuren H (2009). *Agrobacterium*-mediated transformation of friable embryogenic calli and regeneration of transgenic cassava. Nat Protoc.

[CR14] Burns A, Gleadow R, Cliff J, Zacarias A, Cavagnaro T (2010). Cassava: the drought, war and famine crop in a changing world. Sustainability.

[CR15] Buschmann H, Reilly K, Rodriguez MX, Tohme J, Beeching JR (2000). Hydrogen peroxide and flavan-3-ols in storage roots of cassava (*Manihot esculenta* Crantz) during postharvest deterioration. J Agric Food Chem.

[CR16] Buschmann H, Rodriguez MX, Tohme J, Beeching JR (2000). Accumulation of hydroxycoumarins during post-harvest deterioration of tuberous roots of Cassava (*Manihot esculenta* Crantz). Ann Bot.

[CR17] Calvert LA, Thresh JM, Hillcocks RJ, Thresh JM, Bellotti A (2002). Viruses and virus deseases of cassava. Cassava: biology, production and utilization.

[CR18] Chang S, Puryear J, Cairney J (1993). A simple and efficient method for isolating RNA from pine trees. Plant Mol Biol Report.

[CR19] Clough SJ (2005). Floral dip: *agrobacterium*-mediated germ line transformation. Methods Mol Biol.

[CR20] Clough SJ, Bent AF (1998). Floral dip: a simplified method for *Agrobacterium*-mediated transformation of *Arabidopsis thaliana*. Plant J.

[CR21] Cortés DF, Reilly K, Okogbenin E, Beeching JR, Iglesias C, Tohme J (2002). Mapping genes implicated in post-harvest physiological deterioration (PPD) in cassava (*Manihot esculenta* Crantz). Euphytica.

[CR22] De Bolle MFC (2003). Analysis of the influence of promoter elements and a matrix attachment region on the inter-individual variation of transgene expression in populations of *Arabidopsis thaliana*. Plant Sci.

[CR23] El-Sharkawy MA (2004). Cassava biology and physiology. Plant Mol Biol.

[CR24] El-Sharkawy MA (2012). Stress-tolerant cassava: the role of integrative ecophysiology-breeding research in crop improvement. Open J Soil Sci.

[CR25] Fermont AM, van Asten PJA, Tittonell P, van Wijk MT, Giller KE (2009). Closing the cassava yield gap: an analysis from smallholder farms in East Africa. Field Crops Res.

[CR26] Gertz EM, Yu YK, Agarwala R, Schaffer AA, Altschul SF (2006). Composition-based statistics and translated nucleotide searches: improving the TBLASTN module of BLAST. BMC Biol.

[CR27] Gnonlonfin BGJ, Sanni A, Brimer L (2012). Review scopoletin—a coumarin phytoalexin with medicinal properties. Crit Rev Plant Sci.

[CR28] Goodstein DM (2012). Phytozome: a comparative platform for green plant genomics. Nucleic Acids Res.

[CR29] Gutierrez M (1995). Abiotic elicitation of coumarin phytoalexins in sunflower. Phytochemistry.

[CR30] Gutiérrez-Mellado M-C, Edwards R, Tena M, Cabello F, Serghini K, Jorrín J (1996). The production of coumarin phytoalexins in different plant organs of sunflower (*Helianthus annuus* L.). J Plant Physiol.

[CR31] Helliwell C, Waterhouse PM (2003). Constructs and methods for high-throughput gene silencing in plants. Methods.

[CR32] Hillocks RJ, Wydra K, Hillcocks RJ, Thresh JM, Bellotti A (2002). Bacterial, fungal and nematode diseases. Cassava: biology, production and utilization.

[CR33] Hoekema A, Hirsch PR, Hooykaas PJJ, Schilperoort RA (1983). A binary plant vector strategy based on separation of vir- and T-region of the *Agrobacterium tumefaciens* Ti-plasmid. Nature.

[CR34] Jackson AL (2003). Expression profiling reveals off-target gene regulation by RNAi. Nat Biotechnol.

[CR35] Jefferson R, Goldsbrough A, Bevan M (1990). Transcriptional regulation of a patatin-1 gene in potato. Plant Mol Biol.

[CR36] Kai K (2008). Scopoletin is biosynthesized via ortho-hydroxylation of feruloyl CoA by a 2-oxoglutarate-dependent dioxygenase in *Arabidopsis thaliana*. Plant J.

[CR37] Kai K, Shimizu B, Mizutani M, Watanabe K, Sakata K (2006). Accumulation of coumarins in *Arabidopsis thaliana*. Phytochemistry.

[CR38] Karimi M, Inze D, Depicker A (2002). GATEWAY™ vectors for *Agrobacterium*-mediated plant transformation. Trends Plant Sci.

[CR39] Kearse M (2012). Geneious basic: an integrated and extendable desktop software platform for the organization and analysis of sequence data. Bioinformatics.

[CR40] Livak KJ, Schmittgen TD (2001). Analysis of relative gene expression data using real-time quantitative pcr and the 2^– ∆∆Ct^ method. Methods.

[CR41] Morante N (2010). Tolerance to postharvest physiological deterioration in cassava roots. Crop Sci.

[CR42] Moreno I, Gruissem W, Vanderschuren H (2011). Reference genes for reliable potyvirus quantitation in cassava and analysis of cassava brown streak virus load in host varieties. J Virol Methods.

[CR43] Munkacsy G, Sztupinszki Z, Herman P, Ban B, Penzvalto Z, Szarvas N, Gyorffy B (2016). Validation of rnai silencing efficiency using gene array data shows 18.5% failure rate across 429 independent experiments. Mol Ther Nucleic Acids.

[CR44] Nassar N, Vizzotto CS, Schwartz CA, Pires OR (2007). Cassava diversity in Brazil: the case of carotenoid-rich landraces. Genet Mol Res.

[CR45] Owiti J (2011). iTRAQ-based analysis of changes in the cassava root proteome reveals pathways associated with post-harvest physiological deterioration. Plant J.

[CR46] Page M (2009) Modulation of root antioxidant status to delay cassava post-harvest physiological deterioration., University of Bath

[CR47] Prochnik S (2012). The cassava genome: current progress, future directions. Trop Plant Biol.

[CR48] Reilly K, Gómez-Vásquez R, Buschmann H, Tohme J, Beeching JR (2004). Oxidative stress responses during cassava post-harvest physiological deterioration. Plant Mol Biol.

[CR49] Reilly K, Bernal D, Cortes DF, Gomez-Vasquez R, Tohme J, Beeching JR (2007). Towards identifying the full set of genes expressed during cassava post-harvest physiological deterioration. Plant Mol Biol.

[CR50] Sakurai T (2007). Sequencing analysis of 20,000 full-length cDNA clones from cassava reveals lineage specific expansions in gene families related to stress response. BMC Plant Biol.

[CR51] Salehuzzaman SNIM, Jacobsen E, Visser RGF (1993). Isolation and characterization of a cDNA encoding granule-bound starch synthase in cassava (*Manihot esculenta* Crantz) and its antisense expression in potato. Plant Mol Biol.

[CR52] Sánchez T, Dufour D, Moreno JL, Pizarro M, Aragón IJ, Domínguez M, Ceballos H (2013). Changes in extended shelf life of cassava roots during storage in ambient conditions. Postharvest Biol Technol.

[CR53] Sayre R (2011). The BioCassava plus program: biofortification of cassava for sub-Saharan Africa. Annu Rev Plant Biol.

[CR54] Schwab R, Ossowski S, Riester M, Warthmann N, Weigel D (2006). Highly specific gene silencing by artificial microRNAs in *Arabidopsis*. Plant Cell.

[CR55] Uarrota VG, Maraschin M (2015). Metabolomic, enzymatic, and histochemical analyzes of cassava roots during postharvest physiological deterioration. BMC Res Notes.

[CR56] Vanderschuren H (2014). Large-scale proteomics of the cassava storage root and identification of a target gene to reduce postharvest deterioration. Plant Cell.

[CR57] Wesley SV (2001). Construct design for efficient, effective and high-throughput gene silencing in plants. Plant J.

[CR58] Xu J, Duan X, Yang J, Beeching JR, Zhang P (2013). Enhanced reactive oxygen species scavenging by overproduction of superoxide dismutase and catalase delays postharvest physiological deterioration of cassava storage roots. Plant Physiol.

[CR59] Zainuddin IM, Schlegel K, Gruissem W, Vanderschuren H (2012). Robust transformation procedure for the production of transgenic farmer-preferred cassava landraces. Plant Methods.

[CR60] Zidenga T, Leyva-Guerrero E, Moon H, Siritunga D, Sayre R (2012). Extending cassava root shelf life via reduction of reactive oxygen species production. Plant Physiol.

