# Supplementary material for: RNAi inhibition of feruloyl CoA 6′-hydroxylase reduces scopoletin biosynthesis and post-harvest physiological deterioration in cassava (Manihot esculenta Crantz) storage roots
Source: Plant Mol Biol. 2017 Mar 18;94(1):185–95. doi: 10.1007/s11103-017-0602-z (PMC5437147; doi:10.1007/s11103-017-0602-z)
Supplement: Supplementary file 1 — Supplementary material 1 (PPTX 449 KB) [file 11103_2017_602_MOESM1_ESM.pptx]

## Slide 1
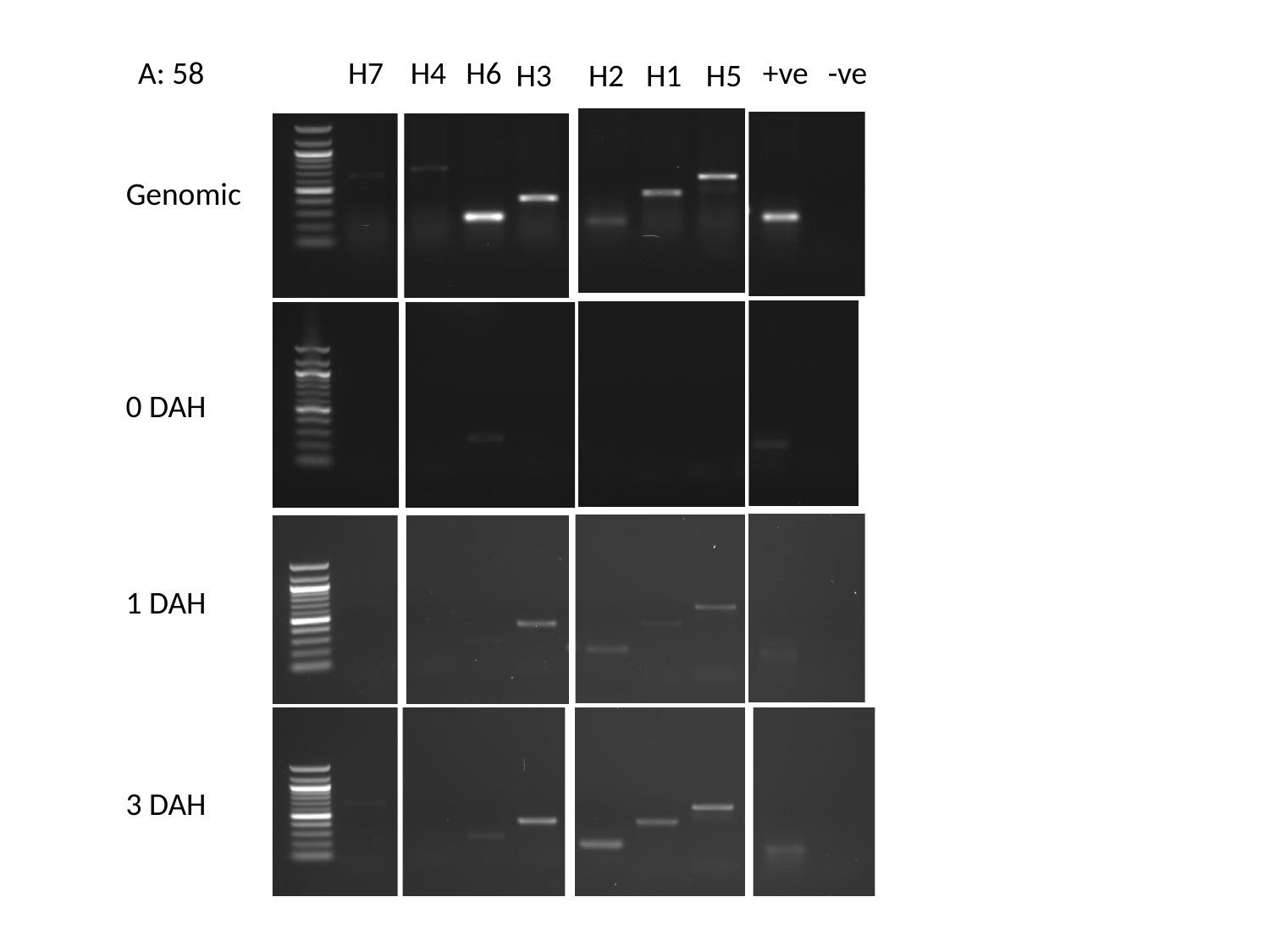

A: 58
H7
H4
H6
+ve
-ve
H2
H3
H1
H5
Genomic
0 DAH
1 DAH
3 DAH

## Slide 2
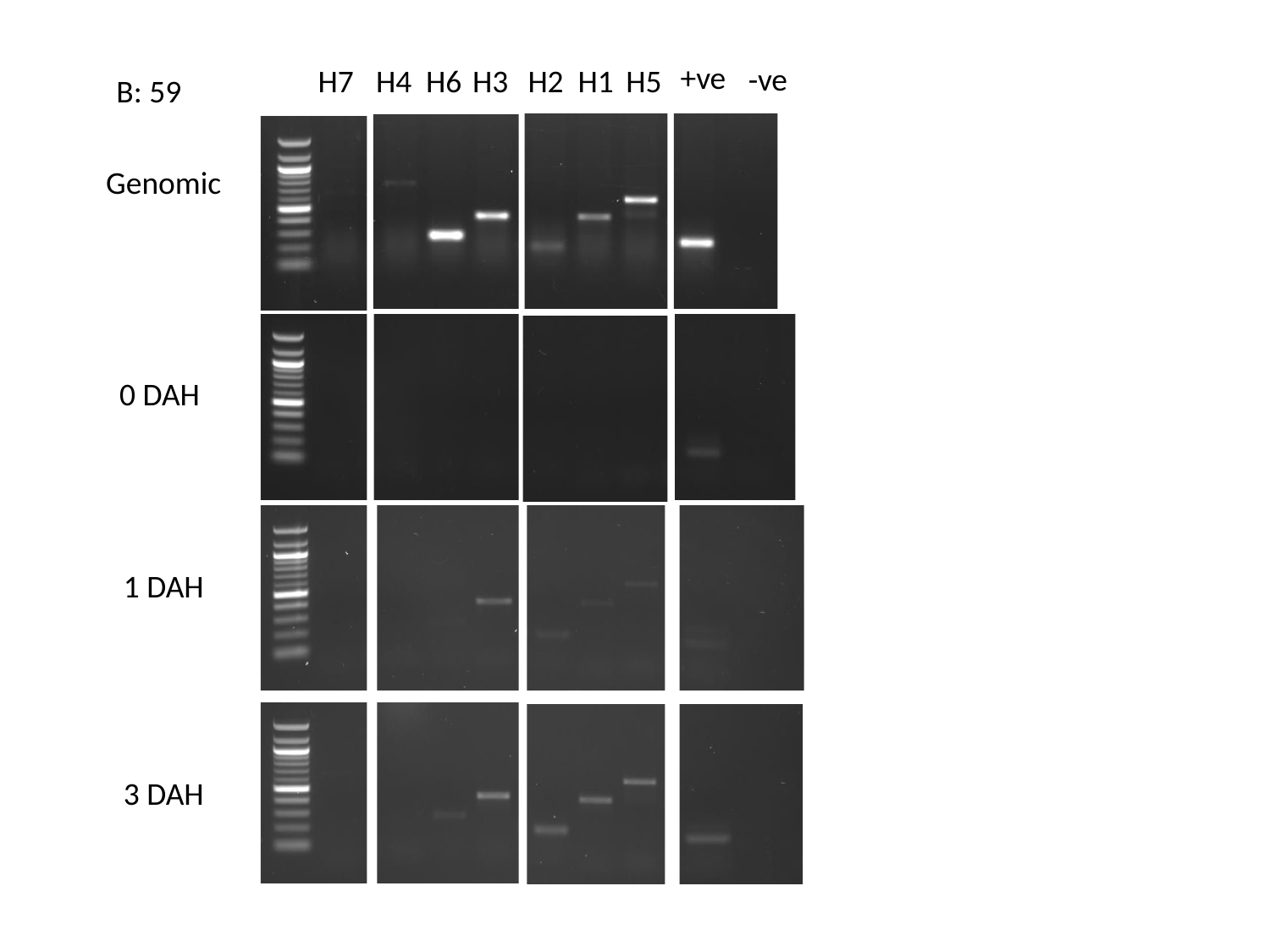

+ve
-ve
H7
H4
H6
H3
H2
H1
H5
B: 59
Genomic
0 DAH
1 DAH
3 DAH

## Slide 3
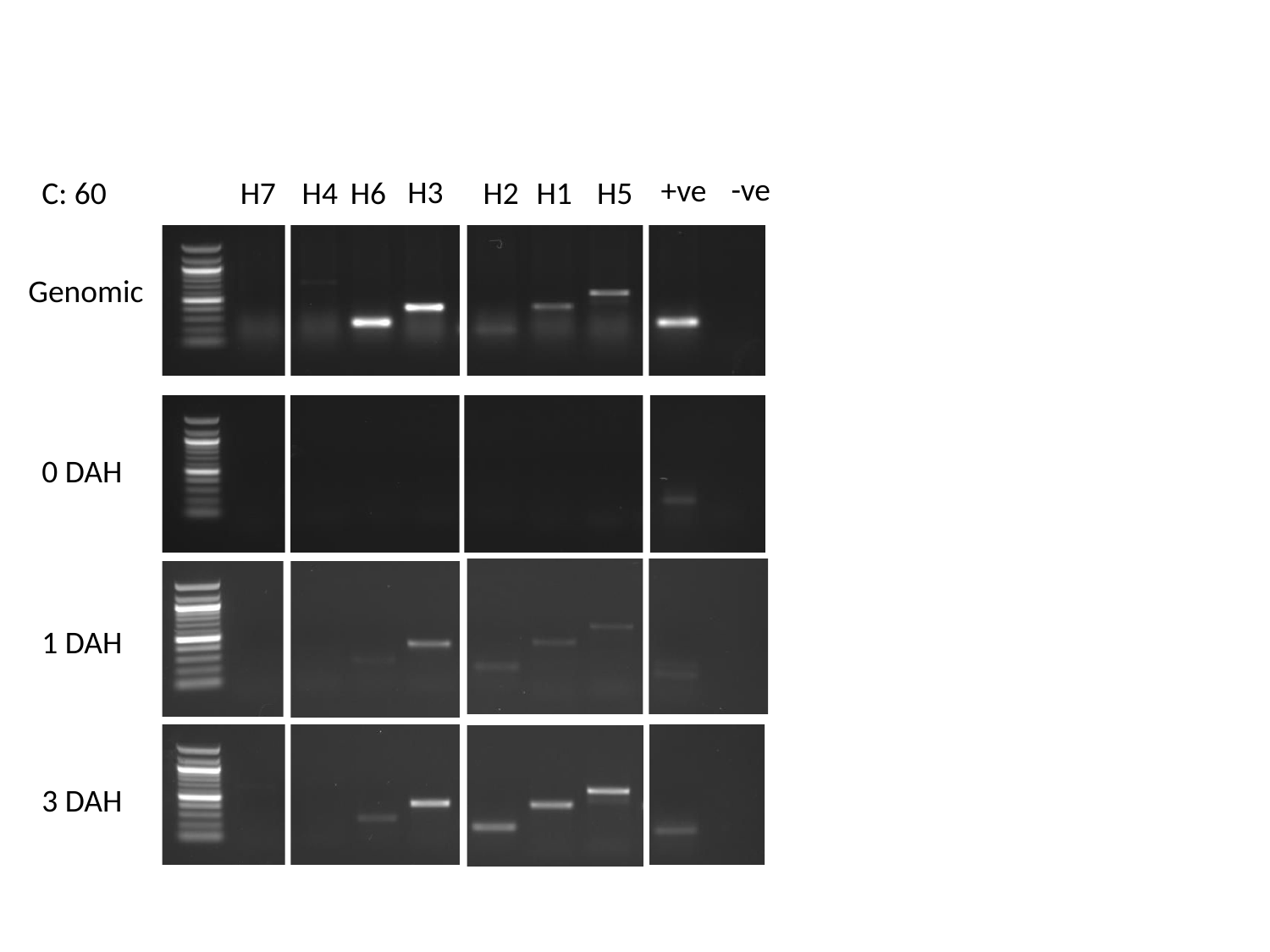

-ve
+ve
H3
H4
H6
H2
H1
H5
C: 60
H7
Genomic
0 DAH
1 DAH
3 DAH

## Slide 4
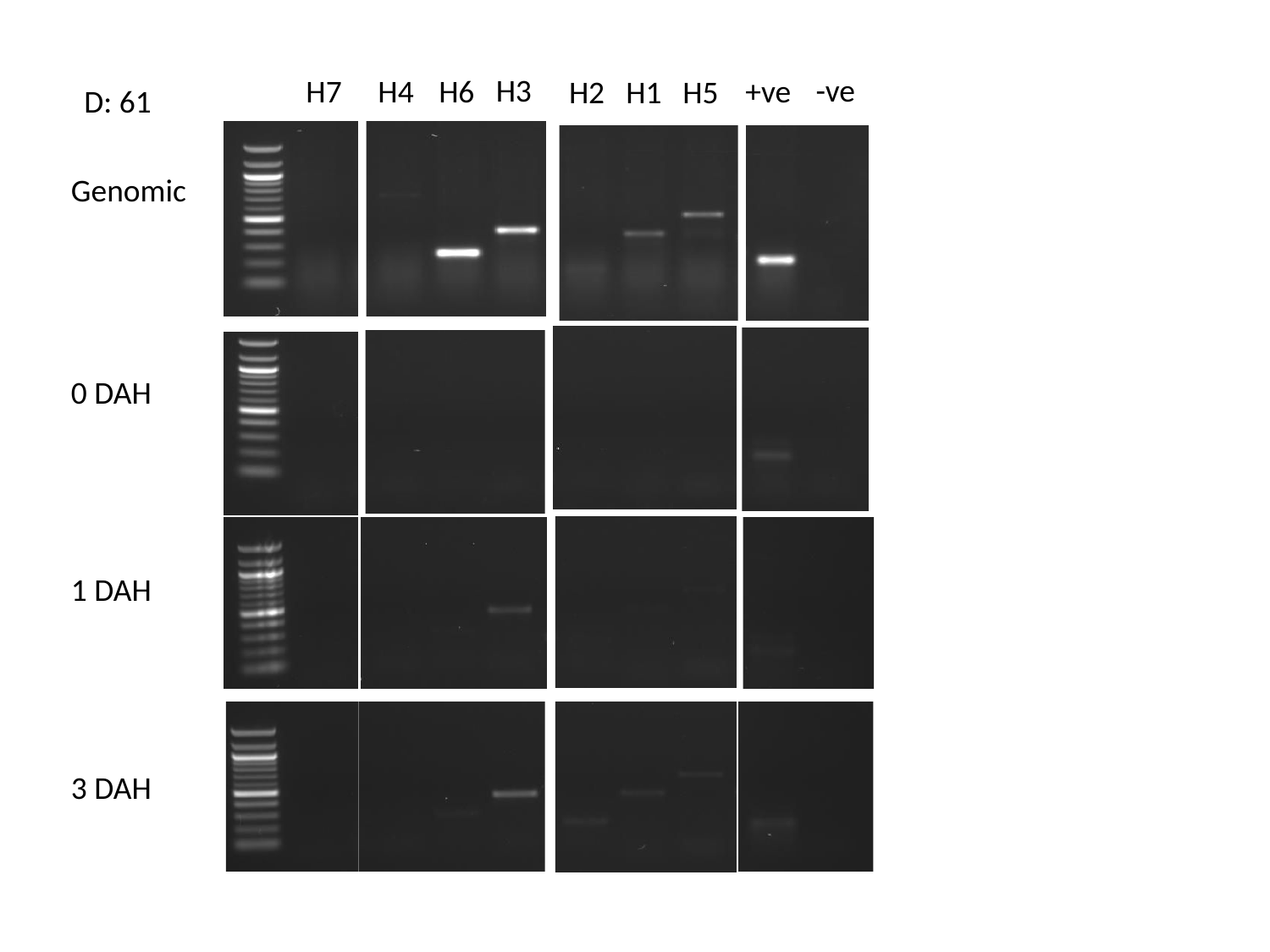

H3
-ve
+ve
H7
H4
H6
H2
H1
H5
D: 61
Genomic
0 DAH
1 DAH
3 DAH

## Slide 5
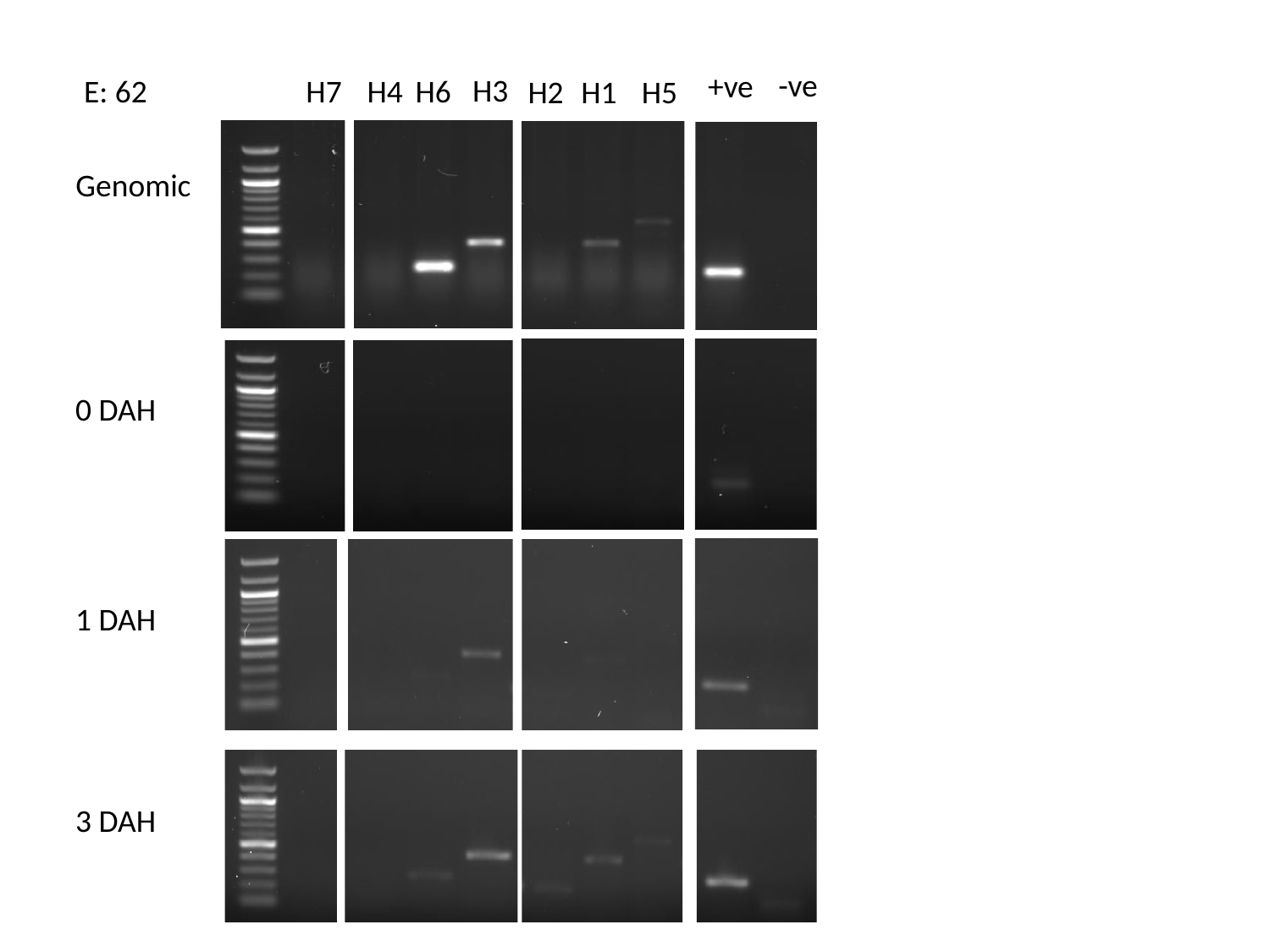

-ve
+ve
H3
E: 62
H7
H4
H6
H2
H1
H5
Genomic
0 DAH
1 DAH
3 DAH

## Slide 6
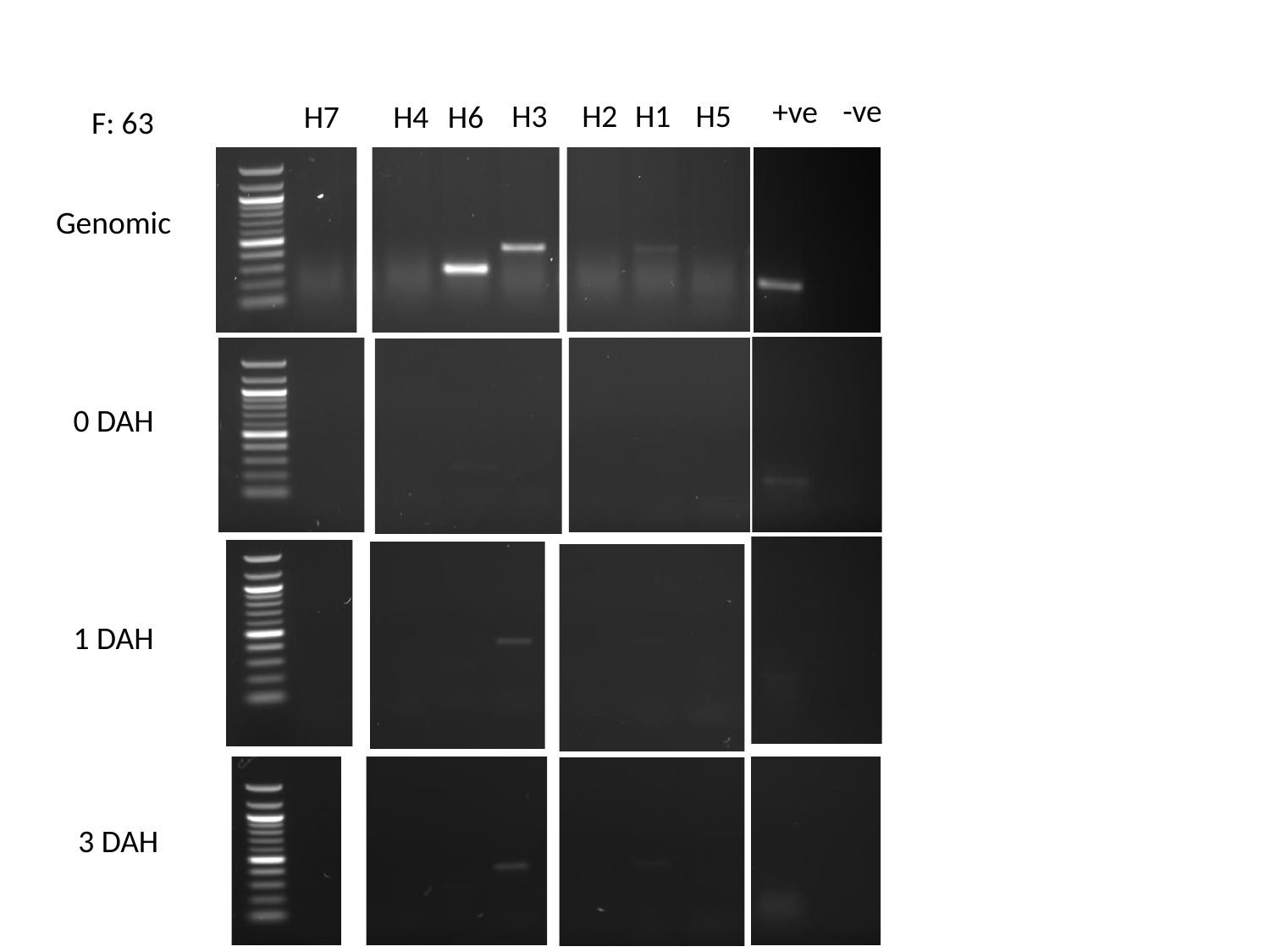

-ve
+ve
H3
H2
H1
H5
H7
H4
H6
F: 63
Genomic
0 DAH
1 DAH
3 DAH

## Slide 7
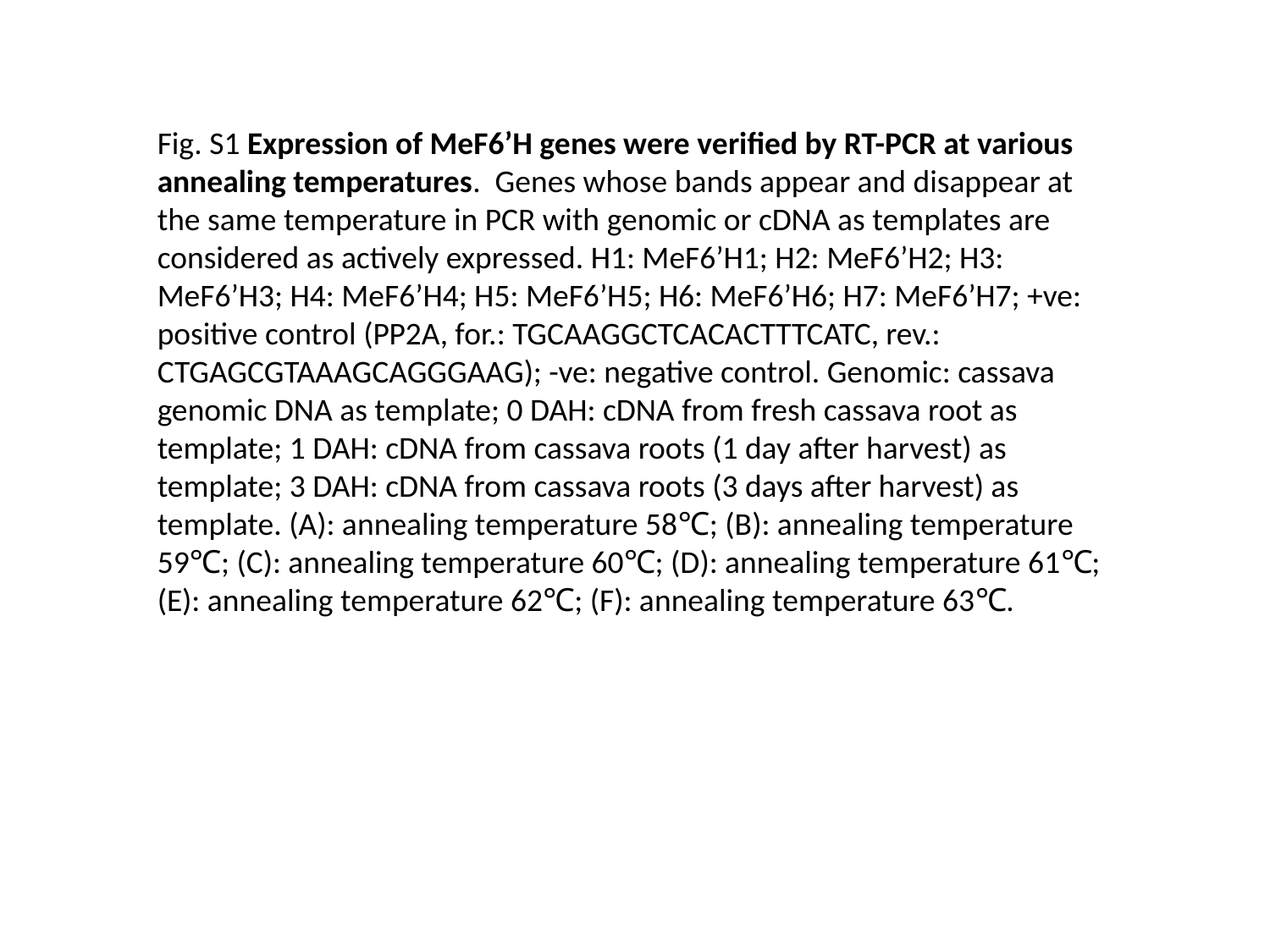

Fig. S1 Expression of MeF6’H genes were verified by RT-PCR at various annealing temperatures. Genes whose bands appear and disappear at the same temperature in PCR with genomic or cDNA as templates are considered as actively expressed. H1: MeF6’H1; H2: MeF6’H2; H3: MeF6’H3; H4: MeF6’H4; H5: MeF6’H5; H6: MeF6’H6; H7: MeF6’H7; +ve: positive control (PP2A, for.: TGCAAGGCTCACACTTTCATC, rev.: CTGAGCGTAAAGCAGGGAAG); -ve: negative control. Genomic: cassava genomic DNA as template; 0 DAH: cDNA from fresh cassava root as template; 1 DAH: cDNA from cassava roots (1 day after harvest) as template; 3 DAH: cDNA from cassava roots (3 days after harvest) as template. (A): annealing temperature 58℃; (B): annealing temperature 59℃; (C): annealing temperature 60℃; (D): annealing temperature 61℃; (E): annealing temperature 62℃; (F): annealing temperature 63℃.
