# Supplementary material for: RNAi inhibition of feruloyl CoA 6′-hydroxylase reduces scopoletin biosynthesis and post-harvest physiological deterioration in cassava (Manihot esculenta Crantz) storage roots
Source: Plant Mol Biol. 2017 Mar 18;94(1):185–95. doi: 10.1007/s11103-017-0602-z (PMC5437147; doi:10.1007/s11103-017-0602-z)
Supplement: Supplementary file 2 — Supplementary material 2 (PPTX 1943 KB) [file 11103_2017_602_MOESM2_ESM.pptx]

## Slide 1
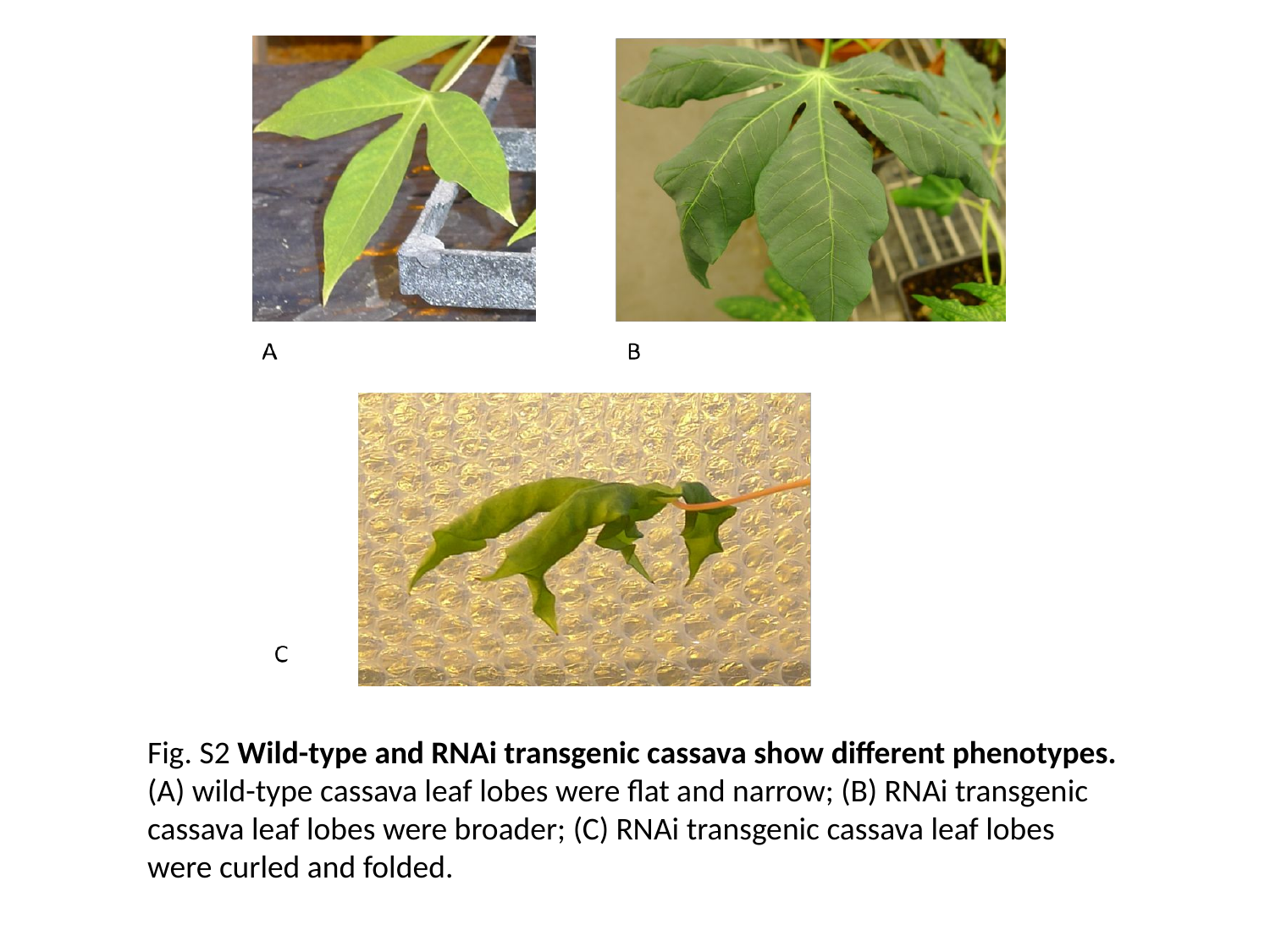

Fig. S2 Wild-type and RNAi transgenic cassava show different phenotypes. (A) wild-type cassava leaf lobes were flat and narrow; (B) RNAi transgenic cassava leaf lobes were broader; (C) RNAi transgenic cassava leaf lobes were curled and folded.
