# Supplementary material for: RNAi inhibition of feruloyl CoA 6′-hydroxylase reduces scopoletin biosynthesis and post-harvest physiological deterioration in cassava (Manihot esculenta Crantz) storage roots
Source: Plant Mol Biol. 2017 Mar 18;94(1):185–95. doi: 10.1007/s11103-017-0602-z (PMC5437147; doi:10.1007/s11103-017-0602-z)
Supplement: Supplementary file 3 — Supplementary material 3 (PPTX 3042 KB) [file 11103_2017_602_MOESM3_ESM.pptx]

## Slide 1
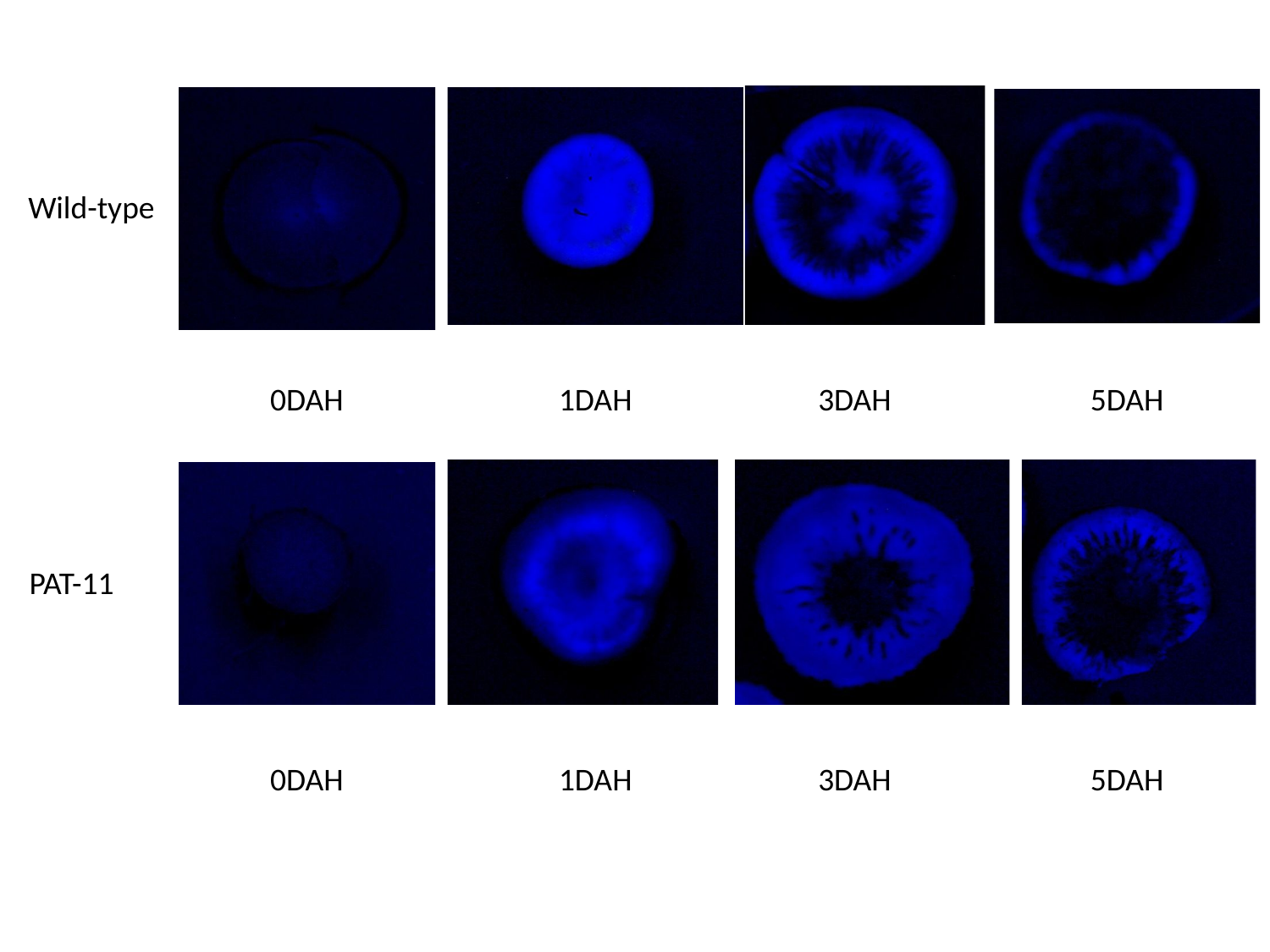

Wild-type
0DAH
1DAH
3DAH
5DAH
PAT-11
0DAH
1DAH
3DAH
5DAH

## Slide 2
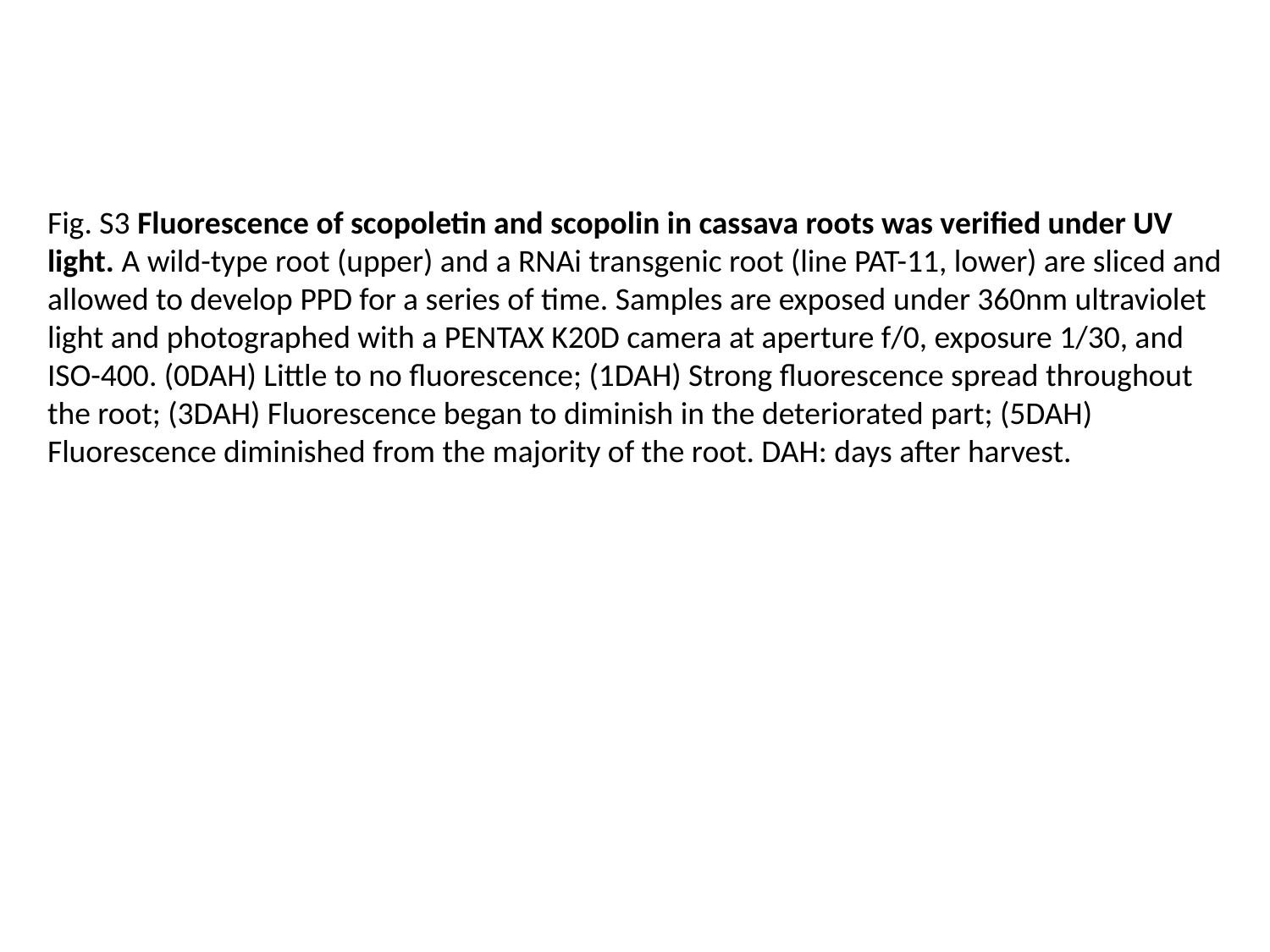

Fig. S3 Fluorescence of scopoletin and scopolin in cassava roots was verified under UV light. A wild-type root (upper) and a RNAi transgenic root (line PAT-11, lower) are sliced and allowed to develop PPD for a series of time. Samples are exposed under 360nm ultraviolet light and photographed with a PENTAX K20D camera at aperture f/0, exposure 1/30, and ISO-400. (0DAH) Little to no fluorescence; (1DAH) Strong fluorescence spread throughout the root; (3DAH) Fluorescence began to diminish in the deteriorated part; (5DAH) Fluorescence diminished from the majority of the root. DAH: days after harvest.
