# Supplementary material for: RNAi inhibition of feruloyl CoA 6′-hydroxylase reduces scopoletin biosynthesis and post-harvest physiological deterioration in cassava (Manihot esculenta Crantz) storage roots
Source: Plant Mol Biol. 2017 Mar 18;94(1):185–95. doi: 10.1007/s11103-017-0602-z (PMC5437147; doi:10.1007/s11103-017-0602-z)
Supplement: Supplementary file 4 — Supplementary material 4 (PPTX 379 KB) [file 11103_2017_602_MOESM4_ESM.pptx]

## Slide 1
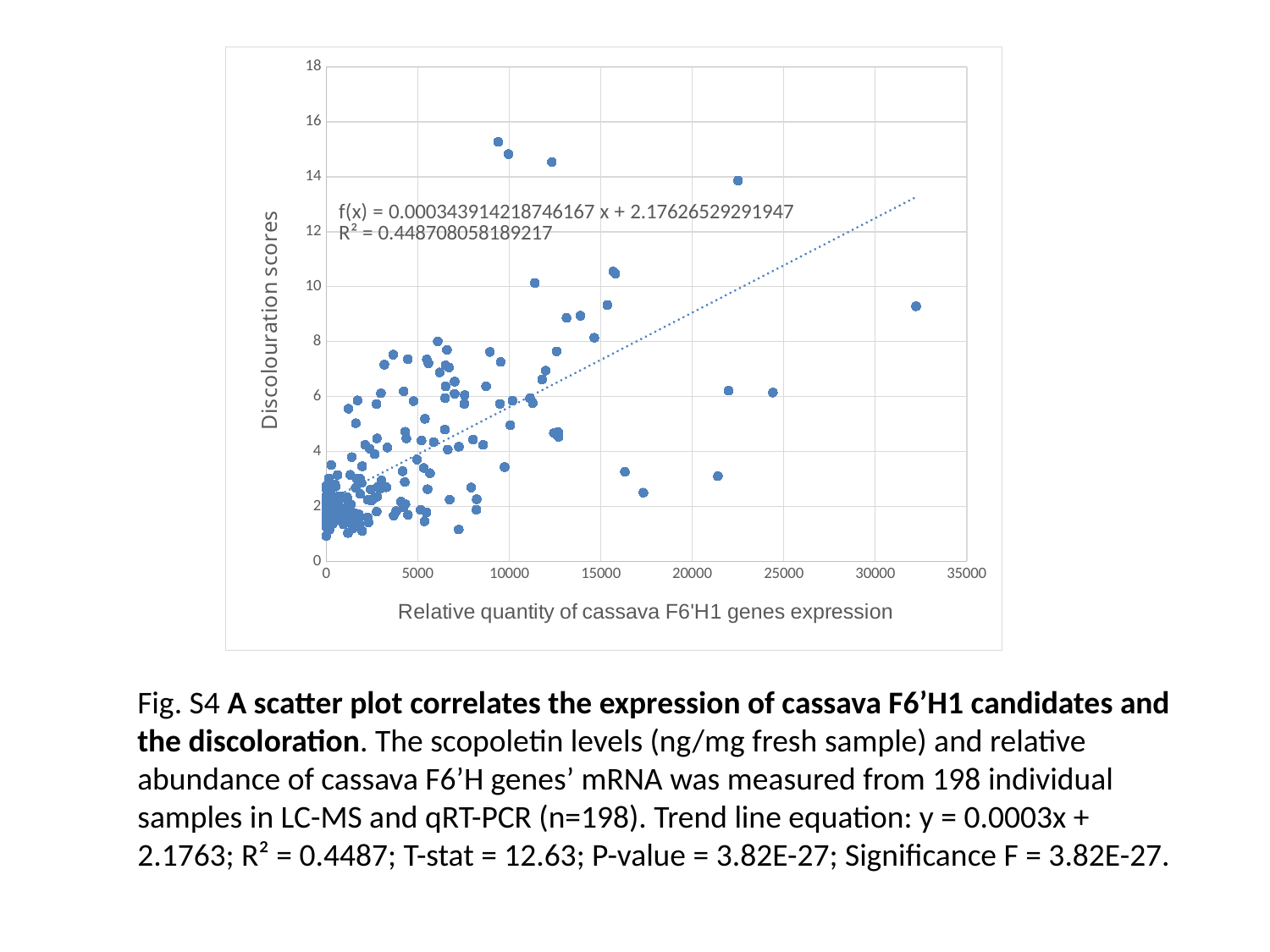

### Chart
| Category | |
|---|---|Fig. S4 A scatter plot correlates the expression of cassava F6’H1 candidates and the discoloration. The scopoletin levels (ng/mg fresh sample) and relative abundance of cassava F6’H genes’ mRNA was measured from 198 individual samples in LC-MS and qRT-PCR (n=198). Trend line equation: y = 0.0003x + 2.1763; R² = 0.4487; T-stat = 12.63; P-value = 3.82E-27; Significance F = 3.82E-27.
