# Supplementary material for: RNAi inhibition of feruloyl CoA 6′-hydroxylase reduces scopoletin biosynthesis and post-harvest physiological deterioration in cassava (Manihot esculenta Crantz) storage roots
Source: Plant Mol Biol. 2017 Mar 18;94(1):185–95. doi: 10.1007/s11103-017-0602-z (PMC5437147; doi:10.1007/s11103-017-0602-z)
Supplement: Supplementary file 6 — Supplementary material 6 (PDF 71 KB) [file 11103_2017_602_MOESM6_ESM.pdf]

```
CLUSTAL O(1.2.3) multiple sequence alignment


MeF6'H_RNAi      ------CCAACACTTGCAGAATCAGCC------ACTGACTCCTTTGATCTCACTGATTTT
MeF6'H1          ATGGCTCCAACAATGGCA------GTATCATCAGCTGATCCCTTTGATCTCACTGATTTT
MeF6'H2          ATGGCTCCAACAATGGCA------GTATCATCAGCTGATTCCTTTGATCTTACTGATTTT
MeF6'H3          ATGGCTCCAACACTTGCAGAATCAGCC------ACTGACTCCTTTGATCTCACTGATTTT
MeF6'H4          ATGGCTCCAGCAATGGCA------TTATCATCCACCGATTCCTTTGATCTCACTGATTTT
MeF6'H5          ATGGCTCCAGCAATGGCA------GTGTCATCCACAGATTCCTTTGATCTCACTGATTTT
MeF6'H6          ATGGCTCCAACACTTGCAGAATCAACCACTGACGCCTCCGCCTTTGATATCGCCGATTTT
MeF6'H7          ATGGCTCCAACAATGGCA------ATATCATCAGCTGATTCCTTTGATCTCACTGATTTT
                       *** ** * ***                *     ******** *  * ******

MeF6'H_RNAi      GTAATCAACCAAGGCAATGGCGTTAAGGGCCTTTCTGATTTGGGCATCAAAAGCCTTCCT
MeF6'H1          GTCATAAACAAAGGGAATGGAGTGAAGGGTCTTTCTGATTTGGGCATCAAAAGCCTTCCT
MeF6'H2          GTCATAAACAAAGGGAATGGAGTGAAGGGTCTTTCTGATTTGGGCATCAAAAGCCTTCCT
MeF6'H3          GTAATCAACCAAGGCAATGGCGTTAAGGGCCTTTCTGATTTGGGCATCAAAAGCCTTCCT
MeF6'H4          GTCATTAACAAAGGCAATGGAGTAAAGGGTCTTTCTGATTTGGGCATCAAAAGCCTTCCC
MeF6'H5          GTCATTAACAAAGGCAATGGAGTAAAGGGTCTTTCTGATTTAGGCATCAAAAGCCTTCCC
MeF6'H6          GTGATCAACCAAGGGAATGGCGTAAAGGGTCTCTCTGAGTTGGGCATCAAAAGCCTTCCT
MeF6'H7          GTCATAAACAAAGGGAATGGAGTGAAGGGTCTTTCTGATTTGGGCATCAAAAGCCTTCCT
                 ** ** *** **** ***** ** ***** ** ***** ** *****************

MeF6'H_RNAi      ATACAATATATTCAACCCCAAGAAGCATTAATCAATATCATCCCTAATGAATCCATACCT
MeF6'H1          TCTCAATATATTCAACCCCAAGAAGCATTGATCAATATCATCCCACAACAATCCATTCCC
MeF6'H2          TCTCAATATATTCAACCCCAAGAAGCATTGATCAATATCATCCCACAACAATCCATTCCC
MeF6'H3          ATACAATATATTCAACCCCAAGAAGCATTAATCAATATCATCCCTAATGAATCCATACCT
MeF6'H4          TCTCAGTATATTCAACCTCAAGAGGCATTGATCAATATCATCCCACAAAAATCTATTCCT
MeF6'H5          TCTCAATATATTCAACCCCAAGAGGCACTTATCAATATCATCCCACAAAAATCTATTCCT
MeF6'H6          CGCCAATATATTCAACCAAAAGAAGCATTGATCAACATCATCCCAAACAAATCCATCCCT
MeF6'H7          TCTCAATATATTCAACCCCAAGAAGCATTGATCAATATCATCCCACAAAAATCCATTCCC
                    ** ***********  **** *** * ***** ********  *  **** ** **

MeF6'H_RNAi      GTCATTGACATGTCAAACTGGGAAAATGATCCTAAGATTGCAGAATCTGTATGTGAAGCT
MeF6'H1          GTCATTGACATGTCTAACTGGGAAACTGATCCCAAAGTCGCTGAATCAGTCTGCGAAGCT
MeF6'H2          GTCATTGACATGTCAAACTGGGAAACTGATCCCAAAGTCGCTGAATCAGTCTGCGAAGCT
MeF6'H3          GTCATTGACATGTCAAACTGGGAAAATGATCCTAAGATTGCAGAATCTGTATGTGAAGCT
MeF6'H4          GTTATTGATATGTCCAACTGGGAAAATGACCCCAGAGTTGCTGAATCAGTCTGTGAAGCT
MeF6'H5          GTTATTGATATGTCCAACTGGGAAAATGACTCCAAAGTTGCTGAATCAGTCTGTGAAGCT
MeF6'H6          GTCATTGACATGGCCAACTGGGAAAATGATCCCAACATCGCTGAATCTGTCTGTGAAGCT
MeF6'H7          GTCATTGACATGTCTAACTGGGAAACTGATCCCAAAGTCGCTGAATCAGTCTGCGAAGCT
                 ** ***** *** * ********** ***  * *   * ** ***** ** ** ******

MeF6'H_RNAi      GCTGAGAGATTTGGATTTTTCCAGCTTGTTAACCATGGAGTTACTCTTGAAGTTCTTGAG
MeF6'H1          GCTCAGCAATTTGGCTTCTTTCAGCTGGTTAACCATGGCGTGCCACTTGAGGTACTCGAT
MeF6'H2          GCTCAGCAATTTGGCTTCTTTCAGCTGGTTAACCATGGCGTGCCACTTGAGGTACTCGAT
MeF6'H3          GCTGAGAGATTTGGATTTTTCCAGCTTGTTAACCATGGAGTTACTCTTGAAGTTCTTGAG
MeF6'H4          GCAGAGAAATTTGGCTTCTTCCAGCTGGTTAACCATGGCGTGCCACTTGAGGTACTCGAT
MeF6'H5          GCAGAGGAATTTGGCTTCTTTCAGTTGGTTAACCATGGCGTGCCACTTGAGGTACTCGAT
MeF6'H6          GCTGAGACATTTGGATTCTTTCAGCTTGTTAACCATGGCGTTCCTCTTGAGGTCCTTGAC
MeF6'H7          GCTCAGCAATTTGGCTTCTTTCAGTTGGTTAACCATGGCGTGCCACTTGAGGTACTCGAT
                 **  **  ****** ** ** *** * *********** **  * ***** ** ** **

MeF6'H_RNAi      GGTGTTGAGGATGCAACTCATGGTTTCTTTGGACTTCCGGCGGCCGTAAAGAGAACATAT
MeF6'H1          GGAGTCAAGGACGCAACACATCGTTTCTTTGGTTTACCAGCAGAGGAGAAGAGGAAATAT
MeF6'H2          GGAGTTAAGGATGCAACGCATCGTTTCTTTGGTTTACCAGCAGAGGAGAAGAGGAAATAT
MeF6'H3          GGTGTTGAGGATGCAACTCATGGTTTCTTTGGACTTCCGGCGGCCGTAAAGAGAACATAT
MeF6'H4          GGCGTTAAGGATGCAACCCATCGGTTCTTTGGGTTGCCAGCAGAGGAGAAGAGGAAGTTC
MeF6'H5          GGCGTTAAGGATGCAACCCATCGGTTCTTTGGGTTGCCAGCAGAGGAGAAGAGAAAGTTC
MeF6'H6          GGTGTTAAAGATGCAACGCATCGTTTCTTTGGGGTTCCAGCTGCTGTGAAGAGGAAGTAT
MeF6'H7          GGAGTTAAGGATGCAACACATCGTTTCTTTGGTTTACCAGCAGAGGAGAAGAGGAAATAT
                 ** **  * ** ***** *** * ********  * ** ** *  *  ***** *  *

MeF6'H_RNAi      TCTAAAGAGCTTTCTCCTTCTAACAGTGTCAGATTTGGCACTAGCTTTAGTCCTGATTCT
MeF6'H1          TCCAAGGAGCTTTCTTCTACTAACAGCGTCAGGTTTGGAACCAGCTTTAGTCCTGATGCA
MeF6'H2          TCCAAGGAGCTTTCTTCTACCAACAGCGTCAGGTTTGGAACCAGCTTTAGTCCTGATGCA
MeF6'H3          TCTAAAGAGCTTTCTCCTTCTAACAGTGTCAGATTTGGCACTAGCTTTAGTCCTGATTCT
MeF6'H4          TCCAAAGAGCTTTCTTCCACCAACAATATCAGATTTGGAACCAGCTTTAGTCCTGATGCA
MeF6'H5          TCCAAAGAGCTTTCTTCCACCAACAACATCAGATTTGGAACCAGCTTTAGTCCTGATGCA
```

```
MeF6'H6         TCTAAGGAGCTTTCGCCTTCTAACAGTGTTAGATTTGGCACCAGCTTTAGCCCTGATTCT
MeF6'H7         TCCAAGGAGCTTTCTTCTACCAACAGCGTCAGGTTTGGAACCAGCTTTAGTCCTGATGCA
                ** ** ********  *  * ****   * ** ***** ** ******** ****** *

MeF6'H_RNAi     GAGAAAGCTCTTGAATGGAAAGACTACCTTAGCCTTTTCTATGTCTCCGACGACGAGGCT
MeF6'H1         GAGAAAGCTCTTGAATGGAAGGATTATCTCAGCCTCTTTTATGTCTCTGAGGAAGAGGCT
MeF6'H2         GAGAAAGCTCTTGAATGGAAGGATTATCTCAGCCTCTTTTATGTCTCTGAGGAAGAGGCT
MeF6'H3         GAGAAAGCTCTTGAATGGAAAGACTACCTTAGCCTTTTCTATGTCTCCGACGACGAGGCT
MeF6'H4         GAGAAAGCTCTTGAATGGAAAGATTACCTTAGCCTCTTCTATGTCTCTGAGGAGGAGGCT
MeF6'H5         GAGAAAGCTCTTGAATGGAAAGATTACCTTAGCCTCTTCTATGTCTCAGAGGAGGAGGCT
MeF6'H6         GAGAAAGCTCTTGAATGGAAAGATTATCTCAGCCTTTTCTATGTCTCTGAGGAAGAAGCT
MeF6'H7         GAGAAGGCTCTTGAATGGAAGGATTATCTCAGCCTCTTTTATGTCTCTGAGGAAGAGGCT
                ***** ************** ** ** ** ***** ** ******** ** ** ** ***

MeF6'H_RNAi     AAT---------------------------------------------------------
MeF6'H1         TTTGCATTATGGCCCAATGCTTGCAGAGATGAAGTTCTTGAATACATGAAGAGATCCCAA
MeF6'H2         TTTGCATTATGGCCTAATGCTTGCAGAGATGAAGTTCTTGAATACATGAAGAGATCCCAA
MeF6'H3         AATGCATTGTGGCCTCCTCAGTGCAAGGATGAATGCTTAGAATACATGAAGAAAGCAGAA
MeF6'H4         TCTGTATTGTGGCCTTCTGCTTGCAGAGATGAAGTCCTTGAATACATGAAGAAATCTCAA
MeF6'H5         TCTGTATTGTGGCCTTCTGCTTGCAGAGATGAAGTCCTTGAATACATGAAGAAATCTCAA
MeF6'H6         TCTGCATTGTGGCCTCCTGAGTGCAAGGATGAATGCTTGGAGTACATGAAGAAATCTGAA
MeF6'H7         TTTGCATTATGGCCTAATGCTTGCAGAGATGAAGTTCTTGAATACATGAAGAGATCCCAA
                  *

MeF6'H_RNAi     ------------------------------------------------------------
MeF6'H1         ATCCTTTGCCGAAAGCTAATGAGTGCTCTCATGGAGAATCTGAACGTGAAGGAAATAGAT
MeF6'H2         ATCCTTTGCCGAAAGCTCATGAGTGCTCTCATGGAGAATCTGAACGTGAAGGAAATAGAT
MeF6'H3         ATTCTATGCAAAAAGCTATTAACTGCCCTAATGGAGAGACTCAATATAAAAGAAATAGAT
MeF6'H4         GTCCTCTGCACGAAGCTAATGACTGCACTCATGGAGAAACTGAACGTAAAAGAAATAGAC
MeF6'H5         GTCCTCTGCACGAAGCTAATGAGTACACTCATGGAGAAACTGAACGTAAAAGAAATAGAC
MeF6'H6         ATCTTATGCAGAAAGCTTTTGACTGCATTAATGGAGAGACTCAATGTAAAAGAAATAGAC
MeF6'H7         ATCCTTTGCCGAAAGCTCATGAATGCTCTCATGGAGAATCTGAACGTGAAGGAAATAGAT


MeF6'H_RNAi     ------------------------------------------------------------
MeF6'H1         GAAACAAAAGAATCTCTTCTAATGGGATCTAAAAGAATCAACCTCAACTACTATCCCAGA
MeF6'H2         GAAACAAAAGAATCTCTTCTAATGGGATCTAAAAGAATCAACCTCAACTACTATCCAAGA
MeF6'H3         GAAAAGAAAGAGTCTCTTTTAATGGGATCAAGAAGAATTAACCTTAACTATTATCCAAGA
MeF6'H4         GAATCCAAAGAATCTCTGCTAATGGGCTCTAAAAGGATTAATCTCAACTATTATCCAAGA
MeF6'H5         GAATCCAAAGAATCTCTGCTAATGGGCTCTAAAAGGATTAATCTCAACTATTATCCAAGA
MeF6'H6         GAGAAGAAAGAGTCTCTTTTAATGGGATCTCGAAGAATCAACCTCAACTACTATCCAAGA
MeF6'H7         GAAACGAAAGAATCCCTTCTAATGGGATCAAAAAGAATTAACCTCAACTATTATCCCAGA


MeF6'H_RNAi     ------------------------------------------------------------
MeF6'H1         TGTCCCAATCCTAATCTTACTGTCGGCGTAGGCCGCCATTCTGATGTTTCATCTCTCACT
MeF6'H2         TGTCCGAATCCTAATCTTACTGTCGGGGTTGGCCGCCATTCTGATGTTTCATCTCTCACT
MeF6'H3         TGTCCAAACCCTCAACTCACCGTCGGGGTTGGCCGCCACTCCGACGTCTCCTCCCTCACT
MeF6'H4         TGTCCAAACCCTCAGCTCACTGTTGGGGTTGGCCGCCACTCCGATGTTTCCTCACTCACT
MeF6'H5         TGTCCAAACCCTCAGCTCACTGTTGGGGTTGGCCGCCACTCCGATGTTTCCTCACTCACT
MeF6'H6         TGTCCAAACCCTCAGCTCACTATCGGTGTCGGCCGCCACTCCGACGTCTCCACCCTCACG
MeF6'H7         TGTCCGAATCCTAATCTTACTGTCGGGGTAGGCCGCCACTCTGATGTTTCATCTCTCACT


MeF6'H_RNAi     ------------------------------------------------------------
MeF6'H1         TTCCTCCTGCAAGACGAAATCGGTGGACTTTACGTCAGAGTGAATGAAGGCAAAGGGGAA
MeF6'H2         TTCCTCCTCCAAGACGAAATTGGTGGACTTTACGTGAGAGTGAATGAAGGCAAAGGGGAA
MeF6'H3         TTTCTCCTCCAAGACGAAATCGGCGGGCTTTACGTGCGAGTGAATGAAGGCAAAGGGGAT
MeF6'H4         TTCCTCCTACAAGACGAAATCGGCGGGCTTTACGTGCGAATCAACGAAGGCAAGGGCGAC
MeF6'H5         TTCCTCCTACAAGACGAAATCGGCGGGCTTTACGTGCGAATCAACGAAGGCAAAGGCGAC
MeF6'H6         TTCCTCCTCCAAGACGAAATCGGTGGACTTTACGTGCGAGTCAACGAAGGAAAAGGAGAA
MeF6'H7         TTCCTCCTGCAAGACGAAATCGGTGGACTTTACGTCAGAGTGAATGAAGGCAAAGGGGAA


MeF6'H_RNAi     ------------------------------------------------------------
MeF6'H1         GAAGATGGCTGGGTTCATGTTCCTCCCATTGAAGGATCTCTTGTGATTAATGTTGGAGAT
MeF6'H2         AAAGATGGCTGGGTTCATGTTCCTCCCATTGAAGGATCTCTTGTGATAAATGTTGGAGAT
MeF6'H3         GAAGATGGTTGGGTTCATGTTCCTCCCATCAAAGGATCTCTTGTGATTAATGTTGGCGAT
MeF6'H4         GAAGATGGCTGGGTTCATGTTCCTCCAATTGAAGGATCCCTTGTGATCAACGTGGGAGAT
```

```
MeF6'H5          GAAGATGGCTGGGTTCATGTTCCTCCTATTGAAGGATCTCTTGTGATCAACGTGGGAGAT
MeF6'H6          GAAGATGGGTGGGTTCATGTTCCTCCGATTGAAGGATCTCTTGTGATAAACGTCGGAGAT
MeF6'H7          GAAGATGGCTGGGTTCATGTTCCTCCCATTGAAGGATCTCTTGTGATTAATGTTGGAGAT


MeF6'H_RNAi      ------------------------------------------------------------
MeF6'H1          GCATTGCAGATACTCAGCAATGGTCGATATAGGAGCGTAGAGCATTGTGTGATTGCAAGT
MeF6'H2          GCATTGCAGATACTCAGCAATGGTCGATATAGGAGCGTAGAGCATTGTGTGATTACAAGT
MeF6'H3          GCATTGCAGATACTCAGCAATGGTCGATATAGGAGTGTAGAGCACTGTGTGATTGCGAGT
MeF6'H4          GCATTGCAGATACTGAGCAATGGTCGATACAAGAGCGTAGAGCACTGTGTGATTGCAAGT
MeF6'H5          GCATTGCAGATACTGAGCAATGGTCGATACAAGAGCGTAGAACACTGTGTGATTGCAAGT
MeF6'H6          GCATTACAAATTCTGAGTAATGGTCGATATAAAAGTATCGAGCATTGTGTGATTGCGAGC
MeF6'H7          GCATTGCAGATACTCAGCAATGGTCGATATAGGAGCGTAGAGCATTGTGTGATTGCAAGT


MeF6'H_RNAi      ------------------------------------------------------------
MeF6'H1          GGAAGCAAGAACAGAATTTCAATCCCCATTTTCGTAAATCCAAAGCCAAGTGATGTGATC
MeF6'H2          GGAAGCAAGAACAGAATTTCAATCCCCATTTTCGTAAATCCAAAGCCAAGTGATGTGATC
MeF6'H3          GGAAGCAAGAATAGAATTTCGATTCCTGTTTTCGTCAATCCAAAGCCTACTGATGTTATC
MeF6'H4          GGGAGCAAGAACAGAATTTCAATCCCCATTTTCGTAAATCCAAAGCCAAATGACGTGATC
MeF6'H5          GGAAGCAAGAACAGAATTTCAATCCCCATTTTCGTAAATCCAAAGCCAAATGATGTGATC
MeF6'H6          GGAAGTAAGAACAGAATTTCTATTCCTGTTTTTGTCAACCCAAAGCCAAGTGATGTGATC
MeF6'H7          GGAAGCAAGAACAGAATTTCAATCCCCATTTTCGTAAATCCAAGGCCAAGTGATGTGATC


MeF6'H_RNAi      ------------------------------------------------------------
MeF6'H1          GGTCCATTGCCTGAACTGATCGCCGCCGGTGAGAAGCCCAAATATAAAGACATTCTTTAT
MeF6'H2          GGTCCATTGCCTGAACTGATCGCCGCCGGAGAGAAGCCGAAATATAAAGATATTCTTTAC
MeF6'H3          GGACCTTTGCCGGAACTGCTTGCCGCCGGAGAGAAGCCTAAATACAAGAACATTTTATAT
MeF6'H4          GGTCCTTTGCCTGAGCTGATCGCTGCCGGAGAGAAGCCTAAATATAAGAACATTCTTTAC
MeF6'H5          GGTCCTTTGCCTGAGCTGATCGCTGCCGGAGAGAAGCCTAAATATAAGAACATTCTTTAC
MeF6'H6          GGACCGTTGCCTGAATTGCTTGCTGCCGGAGAAAAGCCAAAATATAAGAATATTTTATAC
MeF6'H7          GGTCCTTTGCCTGAATTGATCGCCGCCGGAGAGAAGCCCAAATATAAAGACATTCTTTAC


MeF6'H_RNAi      ------------------------------------------------------------
MeF6'H1          TCTGATTATGTCAAGCATTTCTTCAGGAAGGCTCATGATGGCAAGAAGACTGTTGCTTTT
MeF6'H2          TCTGATTACGTCAAGCATTTCTTCAGGAAGGCTCATGATGGCAAGAAGACTGTTGCTTTT
MeF6'H3          TCTGATTATGTGAAGCATTTCTTCAGGAAAGCTCATGACGGAAAGAAGACTGTGGAGTTT
MeF6'H4          TCTGATTATGTGAAGCATTTTTTCAGGAAGGCTCATGATGGCAAGAAGACTGTGGCTTTT
MeF6'H5          TCTGATTATGTGAAGCATTTCTTCAGGAAGGCTCATGATGGCAAGAAGACTGTGGCTTTT
MeF6'H6          TCCGATTATGTGAAGCATTTCTTCAGGAAGGCTCATGATGGGAAGAAGACTGTGGAATTT
MeF6'H7          TCTGATTATGTCAAGCATTTCTTCAGGAAGGCTCACGATGGCAAGAAGACTGTTGCTTTT


MeF6'H_RNAi      ------------------
MeF6'H1          GCTGAGATCTCATCGTAA
MeF6'H2          GCTGAGATCTCATCGTAA
MeF6'H3          GCTGAAGTGTGA------
MeF6'H4          GCTGAAATTTGA------
MeF6'H5          GCTGAGATTTCATCGTAA
MeF6'H6          GCTGAAGTGTAA------
MeF6'H7          GCTGAGATCTAA------
```
