# Supplementary material for: RNAi inhibition of feruloyl CoA 6′-hydroxylase reduces scopoletin biosynthesis and post-harvest physiological deterioration in cassava (Manihot esculenta Crantz) storage roots
Source: Plant Mol Biol. 2017 Mar 18;94(1):185–95. doi: 10.1007/s11103-017-0602-z (PMC5437147; doi:10.1007/s11103-017-0602-z)
Supplement: Supplementary file 7 — Supplementary material 7 (DOCX 20 KB) [file 11103_2017_602_MOESM7_ESM.docx]

Table S1 **Percentage similarity between Arabidopsis and cassava F6’H genes’ cDNA sequences.**

| **Name of genes** | **AtF6'H1** | **AtF6'H2** | **MeF6'H1** | **MeF6'H2** | **MeF6'H3** | **MeF6'H4** | **MeF6'H5** | **MeF6'H6** | **MeF6'H7** |
| --- | --- | --- | --- | --- | --- | --- | --- | --- | --- |
| **AtF6'H1** | 100.00 | 75.72 | 66.57 | 66.76 | 66.10 | 67.04 | 67.23 | 66.48 | 66.95 |
| **AtF6'H2** | 75.72 | 65.16 | 100.00 | 97.80 | 82.31 | 88.21 | 88.64 | 81.95 | 97.61 |
| **MeF6'H1** | 66.57 | 65.16 | 100.00 | 97.80 | 82.31 | 88.21 | 88.64 | 81.95 | 97.61 |
| **MeF6'H2** | 66.76 | 65.26 | 97.80 | 100.00 | 82.41 | 88.58 | 89.01 | 82.41 | 97.24 |
| **MeF6'H3** | 66.10 | 64.13 | 82.31 | 82.41 | 100.00 | 82.78 | 82.59 | 86.65 | 82.50 |
| **MeF6'H4** | 67.04 | 65.82 | 88.21 | 88.58 | 82.78 | 100.00 | 97.51 | 82.50 | 88.67 |
| **MeF6'H5** | 67.23 | 66.20 | 88.64 | 89.01 | 82.59 | 97.51 | 100.00 | 81.95 | 88.95 |
| **MeF6'H6** | 66.48 | 64.43 | 81.95 | 82.41 | 86.65 | 82.50 | 81.95 | 100.00 | 82.14 |
| **MeF6'H7** | 66.95 | 64.98 | 97.61 | 97.24 | 82.50 | 88.67 | 88.95 | 82.14 | 100.00 |

Table S2 **Percentage similarity between Arabidopsis and cassava F6’H genes’ amino acid sequences.**

| **Name of genes** | **AtF6'H1** | **AtF6'H2** | **MeF6'H1** | **MeF6'H2** | **MeF6'H3** | **MeF6'H4** | **MeF6'H5** | **MeF6'H6** | **MeF6'H7** |
| --- | --- | --- | --- | --- | --- | --- | --- | --- | --- |
| **AtF6'H1** | 100.00 | 76.39 | 68.36 | 67.80 | 64.12 | 68.08 | 67.51 | 64.89 | 67.80 |
| **AtF6'H2** | 76.39 | 100.00 | 67.51 | 67.23 | 64.97 | 67.80 | 68.08 | 64.04 | 67.23 |
| **MeF6'H1** | 68.36 | 67.51 | 100.00 | 99.17 | 86.91 | 92.52 | 92.84 | 86.98 | 98.61 |
| **MeF6'H2** | 67.80 | 67.23 | 99.17 | 100.00 | 86.63 | 92.24 | 92.56 | 86.43 | 98.34 |
| **MeF6'H3** | 64.12 | 64.97 | 86.91 | 86.63 | 100.00 | 86.63 | 86.07 | 91.14 | 86.91 |
| **MeF6'H4** | 68.08 | 67.80 | 92.52 | 92.24 | 86.63 | 100.00 | 98.34 | 86.43 | 92.80 |
| **MeF6'H5** | 67.51 | 68.08 | 92.84 | 92.56 | 86.07 | 98.34 | 100.00 | 85.60 | 92.52 |
| **MeF6'H6** | 64.89 | 64.04 | 86.98 | 86.43 | 91.14 | 86.43 | 85.60 | 100.00 | 86.98 |
| **MeF6'H7** | 67.80 | 67.23 | 98.61 | 98.34 | 86.91 | 92.80 | 92.53 | 86.98 | 100.00 |
